# Navigating the DNA encoded libraries chemical space

**DOI:** 10.1038/s42004-020-00374-1

**Published:** 2020-09-11

**Authors:** Alfredo Martín, Christos A. Nicolaou, Miguel A. Toledo

**Affiliations:** 1 Eli Lilly and Company, Avda. de la Industria, 30, 28108 Alcobendas, Madrid Spain; 2 Discovery Chemistry, Lilly Research Laboratories, Eli Lilly and Company, Indianapolis, IN 46285 USA

**Keywords:** Chemical libraries, Cheminformatics

## Abstract

DNA-encoded library (DEL) technology is a novel ligand identification strategy that allows the synthesis and screening of unprecedented chemical diversity more efficiently than conventional methods. However, no reports have been published to systematically study how to increase the diversity and improve the molecular property space that can be covered with DEL. This report describes the development and application of eDESIGNER, an algorithm that comprehensively generates all possible library designs, enumerates and profiles samples from each library and evaluates them to select the libraries to be synthesized. This tool utilizes suitable on-DNA chemistries and available building blocks to design and identify libraries with a pre-defined molecular weight distribution and maximal diversity compared with compound collections from other sources.

## Abstract (web summary)

DNA-encoded libraries offer a powerful method for identifying small molecules with valuable functions, but efficiently designing libraries is a challenge. Here an algorithm called eDESIGNER is used to systematically screen and identify libraries with maximal structural diversity.

## Introduction

DNA-encoded libraries (DEL) technology is a game changing innovation in drug discovery. Conceptually designed by Brenner and Lerner^1^, DEL allows the synthesis and screening of millions, or even billions, of encoded compounds cheaper and quicker than using conventional methods^2^. This technology connects the disciplines of molecular biology and organic chemistry through the use of synthetic chemistry cycles to introduce diverse small molecule building blocks (BBs) encoded by unique DNA tags. Several cycles of affinity selection, typically involving an immobilized target protein and a pool of libraries, yield a mixture of compounds enriched in binders to the protein of interest. Amplification of the DNA region by polymer chain reaction methods and posterior next generation sequencing permits the identification of the structure of the binding molecules^3–8^.

The progress achieved in this field during the last two decades has transformed DEL to a powerful production tool for most pharmaceutical companies to identify new ligands for both novel and traditional biological targets^2–5,8–13^. Despite of this, a remaining challenge for DEL is achieving the right balance of library size and molecular properties. This would facilitate the use of DEL actives as medicinal chemistry starting points.

Three main factors play a critical role in the quality and diversity of a DEL collection of compounds: variety of reliable chemistries that are DNA compatible, accessibility to a diverse and large set of BBs, and experience of the designer. Development of new reactions and incorporation of new BBs have been an active area in the past recent years^5,7,13–16^. In contrast, DEL library design and selection, and DEL chemical space overall, has received much less attention^9,17^. Recently, Pfizer^18^ reported an algorithm to optimize properties of libraries by selecting a subset of monomers to be used within a specific design.

A brute force approach to fully explore DEL chemical space could utilize all DNA-amenable reactions and available BBs to comprehensively describe possible DEL library designs through a combinatorial process. Each design could then be used to enumerate all possible library compounds. The most desirable libraries could then be selected via analyzing the properties of the enumerated compounds. This approach, albeit conceptually sound, is impractical due to the very large numbers of virtual compounds possible, well beyond current computational capabilities.

In order to address this combinatorial explosion problem, we developed eDESIGNER (https://github.com/jamflcgh/edesigner_core) that employs a staged approach relying on designing, sampling, and profiling to propose libraries with increased overall high-quality potential. Initially, eDESIGNER prepares all theoretically feasible DEL designs using known amenable chemistry and existing BBs. It then applies constraint rules at the library design level, focusing on restricting heavy atom count distribution while maintaining a minimum library size. Once the designs are created, a random sampling approach is employed to prepare representative subsets subsequently used to evaluate the quality and diversity of each library within a reasonable and still practical computational depth^19^. This approach enables cheminformatics analysis of the proximal DEL library space to obtain conclusions that approximate full enumeration and are valuable for the purpose of DEL design prioritization.

To the best of our knowledge, no similar reports have been published to systematically study DEL space and improve the properties of production libraries.

## Results

**Fig. 1:**
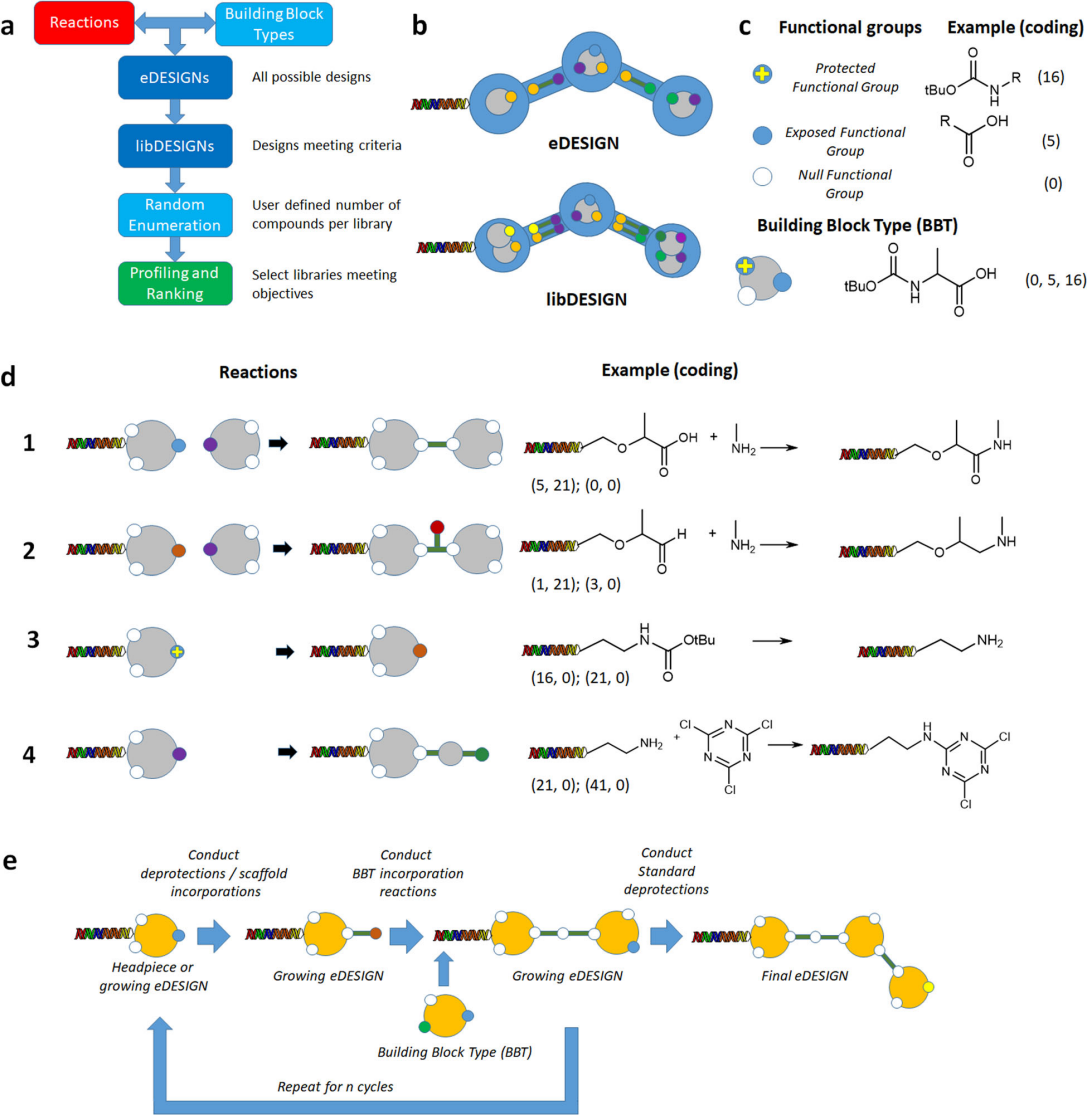
eDESIGNER concepts. **a** Description of the overall eDESIGNER process: reactions and building block types (BBTs) are combined to generate all possible eDESIGNs, then eDESIGNs are merged into libDESIGNS when they can be implemented together in the same experimental conditions. Only libDESIGNs meeting specific criteria survive and their diversity is analyzed through a sample of 10,000 compounds per libDESIGN. **b** eDESIGN and libDESIGN objects: BBTs and reactions are combined in graph objects in which edges represent reactions and nodes are BBTs. libDESIGNS combine several eDESIGNS with the same topology and compatible reactions. **c** Functional group (FG) and BBT objects: a functional FG is a handle that can be used to link BBTs through reactions. Some FGs are exposed for reactions, and some are protected and need deprotection reactions to become exposed. The null FG is added to simplify the code. A combination of FGs defines a BBT. **d** Reaction object: connecting reactions connect two FGs to link two BBTs eliminating the FGs used (entry 1) or creating a new one (entry 2). Deprotection reactions transform an FG into a different one without addition of mass (entry 3) or incorporating a scaffold (entry 4). **e** Iterative process to generate an eDESIGN: a growing eDESIGN, characterized by its list of FGs, can then further incorporate new BBTs using additional reactions, convert protected FGs in exposed FGs, or add a non-coded scaffold.

eDESIGNER relies on the encoding of a reaction system that is capable to enumerate multi-step synthesis on DNA (Fig. 1). A fundamental unit of our process is a functional group (FG), i.e., the structural handle to connect BBs and construct molecules (Fig. 1c). Our definition of FG is not the standard one used in organic chemistry. We define the FG as a group of atoms that is able to participate in a reaction amenable to on-DNA synthesis, or a group of atoms that mask an FG as defined previously, and can be unmasked with conditions compatible with the presence of DNA. We code the FG object using an integer identifier. In some instances a classical FG (e.g., the amide) is not in our list of FGs, while in others, the FG of atoms could represent a more complex entity, such as ortho-fluoronitroarene, because the fluoroarene and the nitro groups in ortho position act in coordination to construct the benzimidazole ring in one of our DNA-amenable reactions. Examples of the FGs and on-DNA synthetic reactions utilized in this study can be found in  Supplementary Method 1 and Table 1 and Supplementary Method 1 and Table 2 respectively. All FGs contain either a reactive group or a group that protects a reactive group that is uncovered under specific experimental conditions. A building block type (BBT) is defined as a combination of exactly three FGs (including the null FG) and is coded as a tuple of three integers corresponding to its FGs. The number of FGs in the BBT, and the inclusion of the null FG, has been introduced to simplify the eDESIGNER algorithm implementation. In this setting, a BBT with two null FGs is regarded as monofunctional, with one null FG as bifunctional, and with no null FGs as trifunctional. The BBT defines a group of BBs that can be treated as identical in terms of reactivity since they have the same combination of FGs. Within the eDESIGNER framework, BBTs (not individual BBs) are the units that comprise library designs. This BBT scheme accounts for mono-, bi-, and tri-functional BBs used in our designs.

**Table 1:** Number of eDESIGNs, libDESIGNs, and selected libDESIGNs.

Number of cycles	Reaction scope	Minimum library size	Target median number atoms	Number of eDESIGNS	Number of libDesigns	Number of selected libDesigns
2	Production	50,000	29	257,768	1207	274
2	Both	50,000	29	467,890	3373	642
3	Production	1,000,000	29	23,512,276	58,765	762
3	Both	1,000,000	29	64,454,231	266,552	2916

**Table 2:** Summary of building block and building block type distribution.

	All sources		Lilly internal		Commercial	
	BBTs	BBs	BBTs	BBs	BBTs	BBs
Monofunctional	42	227,322	42	96,871	42	130,451
Bifunctional	355	70,279	287	25,691	321	44,588
Trifunctional	469	6376	306	2313	391	4063

The last piece necessary to complete an eDESIGN is the reaction (Fig. 1d). We defined reactions as objects that operate on FGs, either to connect a BBT to a growing eDESIGN by linking two of their FGs (coupling reactions), or to transform a specific FG to a different one in a growing eDESIGN (deprotection reactions). We code reactions as a pair of tuples, the first containing the index of the FGs producing the reaction and the second, the FGs that are generated by the reaction.

Connecting reactions use an exposed FG from a BBT and an FG from a growing eDESIGN to connect the two entities. The resulting eDESIGN inherits all the FGs from the previous eDESIGN and the incoming BBT with the exception of the ones participating in the reaction. The latter are replaced by the output of the reaction as additional FGs added to the eDESIGN (Fig. 1d, entries 1 and 2).

In our definition, deprotection reactions act on an FG from a growing design and transform it into a different one, that can be employed by a connecting reaction, with or without the addition of extra atoms. Thus, the central definition of a deprotection reaction is that the transformation is not coded on the DNA. If the deprotection reaction adds mass to the eDESIGN, this additional mass is regarded as a scaffold (e.g., reacting a primary amine with cyanuric chloride converts the primary amine FG into a dichlorotriazine FG as in Fig. 1d, entry 4). In other cases, the deprotection reaction simply uncovers a protected FG, for example, removing the BOC protecting group from a tert-butoxycarbamate as in Fig. 1d, entry 3. The two types of deprotections are encoded in identical manner by the eDESIGNER implementation. The list of deprotection reactions used by eDESIGNER is available in Supplementary Method 1 and Supplementary Table 3.

The incorporation of BBTs is performed in an iterative fashion for a predefined number of cycles to create an eDESIGN (Fig. 1e). The final step in the creation of library designs is the combination of eDESIGNS into another structure called libDESIGN. A libDESIGN shares the same topological arrangement with an eDESIGN but can hold more than one BBT in each node and more than one reaction in each edge if BBTs and reactions are compatible within the experimental conditions required to build the library.

We run eDESIGNER using three BB sources for the purposes of the work presented in this manuscript. The sources included Lilly’s internal collection and BBs readily available from Enamine and Sigma. Externally available BBs were combined and treated as a single source referred to as *External*. In total 303,977 BBs were used in this work (both internal and external), however, all restrictions in terms of library size and heavy atom count per libDESIGN in this study were calculated based solely on internal BBs.

Two categories of reactions were defined for this study: the first contained reactions previously used in a library production at Lilly (PRD), while the second also included reactions experimentally validated, but not yet utilized in production when preparing this manuscript (BOTH).

We performed four eDESIGNER runs varying the number of cycles (2 or 3) and the set of reactions (in production or both in production and validation). Table 1 summarizes the number of library designs obtained in each experiment together with some important parameters.

The minimum library size parameter sets the lower threshold to the number of final compounds in a library prepared only with internal BBs. The number of atoms accounted as incorporated from the headpiece was set to 4. For all designs, the median number of atoms was set to 29. This target was established to match the heavy atom count of our screening collection (Lilly Diversity Cassette (LDC)), considering the extra four-heavy atoms added by the DNA headpiece.

As shown in Table 1, the number of eDESIGNS is very large, especially for the three-cycle libraries using both reaction types where they reach 64 million. The number of libDESIGNs is drastically reduced (in the case of three-cycle designs with all reactions from 64 million eDESIGNs to 266,552 libDESIGNs). This set is further narrowed to 2916 that could generate enough molecules (more than one million) while simultaneously maintaining a molecular size distribution with a median of 29 atoms at the same time.

**Fig. 2:**
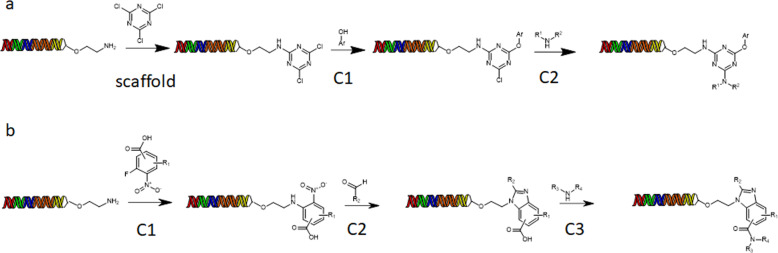
eDESIGNER design examples. **a** 2-cycle libDESIGN. **b** 3-cycle libDESIGN.

Figure 2 shows two representative libDESIGNS corresponding to a two- and three-cycle libraries (panels a and b, respectively). The two-cycle library comprises the introduction of the triazine scaffold followed by a nucleophilic aromatic substitution with phenols and finally another nucleophilic aromatic substitution with amines giving rise to a potential library of 2,584,050 members meeting the predefined heavy atom distribution criteria. The three-cycle library in panel b starts with a nucleophilic aromatic substitution of a series of ortho-fluoronitroarenes containing a carboxylic acid FG, followed by imidazole formation with aldehydes and amide formation with amines. This design could generate a library of 41,374,476 members maintaining the predefined heavy atom distribution. The configuration instruction files for the enumeration of compounds for both libDESIGNS are available in Supplementary Figs. 8 and 9.

**Fig. 3:**
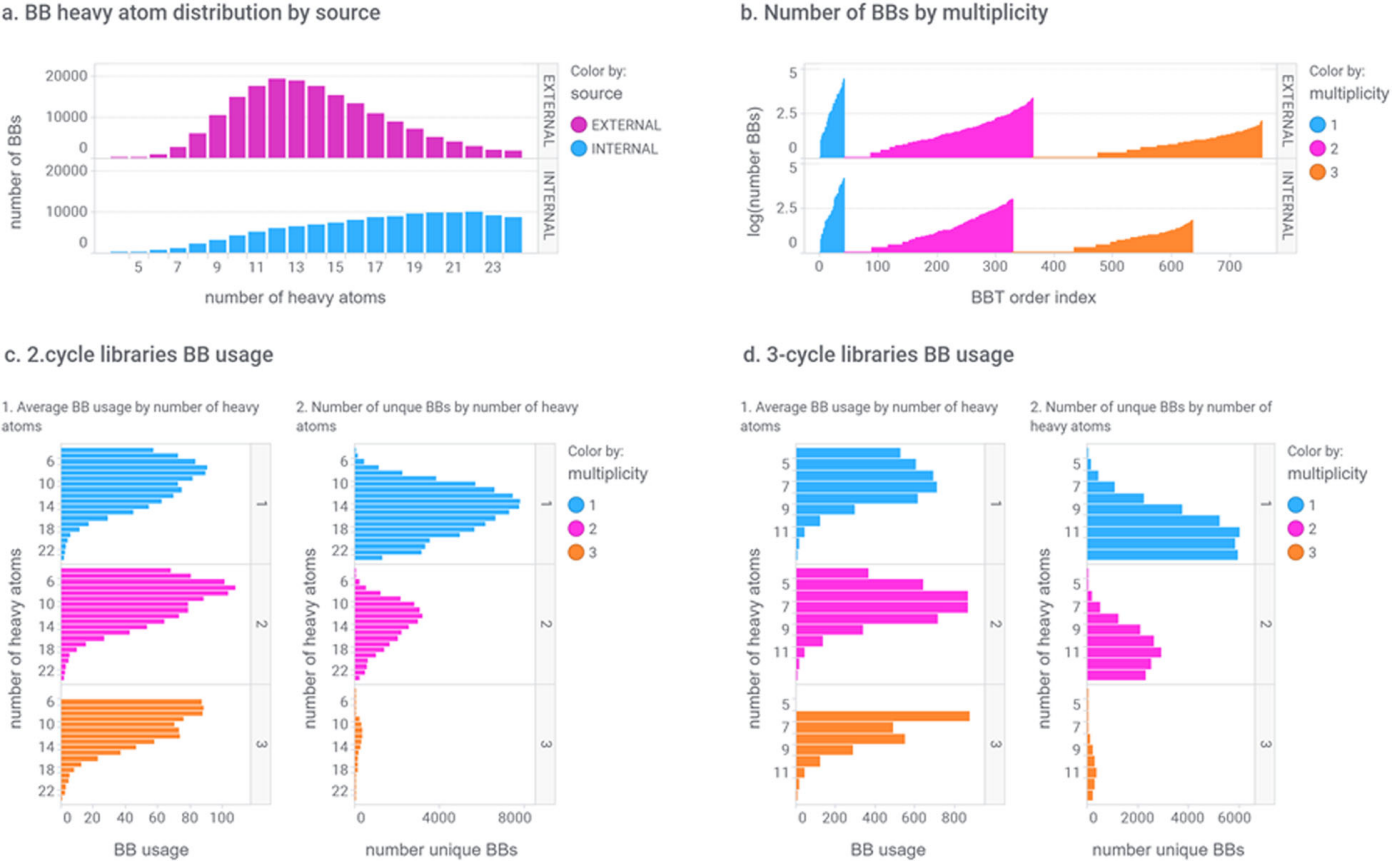
Building block analysis. **a** Effective heavy atom distribution of BBs by source (magenta: external BBs, cyan: internal BBs). **b** Number of building blocks with respect to the multiplicity in their functionality (cyan: monofunctional, magenta: bifunctional, orange: trifunctional). **c** Analysis of BBs used by eDESIGNER in the two-cycle library designs by BB multiplicity. **d** Analysis of BBs used by eDESIGNER in the three-cycle library designs by BB multiplicity.

BBs were classified with regard to their BBT assignment and annotated by source and effective number of atoms (how many atoms they incorporate to the final compound). In the run reported here, 303,977 BBs were identified belonging to any of the valid BBTs. Panel a of Fig. 3 presents the effective heavy atom distribution of these BBs by source. Lilly’s internal BBs tend to be heavier, probably because they tend to be more elaborated as they come mainly from SAR campaigns of different projects. Figure 3b and Table 2 show the BBs used in this study with respect to the multiplicity in their functionality. The data indicate that the number of possible bifunctional and trifunctional BBTs is larger than monofunctional ones due to combinatorial effects. However, the number of BBs in each BBT is decreasing when the multiplicity in their functionality is increasing as expected because of the increased complexity. This analysis also shows that vendors and Lilly’s BB collections have similar profile in terms of multiplicity.

A detailed analysis on BBs was performed to identify those most frequently used by eDESIGNER to generate two- and three-cycle libraries. Results of this analysis are summarized in Fig. 3c, d.

In order to build the 642 two-cycle (panel c) or the 2916 three-cycle library designs (panel d) using all reactions, eDESIGNER utilized 232,254 and 92,909 BBs, respectively, from the set of 303,977 that were assigned to any of the BBTs. On average, each BB is used 46 or 335 times. Chart 1 in panels c and d (Fig. 3) shows the distribution of the average number of times each individual BB is used with respect to its number of atoms. Chart 2 in panels c and d depicts the number of unique BBs with a specific number of atoms that were used in the libraries. Color is used in all diagrams to indicate the multiplicity of BBTs. It is worth pointing out that this information has proven extremely valuable to identify the most versatile and high-impact BBs to prioritize purchasing campaigns to enrich our BB collection.

This information also underlines that three-cycle libraries are more restrictive in the selection of BBs to meet the established limits on the distribution of the number of atoms of the final product compounds. According to the data, trifunctional BBs are much more frequently used than it would be expected based on their relative number in the collection of available BBs. The same is true, albeit to lower extent, for bifunctional BBs. This can be rationalized because trifunctional BBs add more topological diversity in libraries and therefore can be employed in more different ways. The data also show that low molecular weight BBs, although limited in number, are used more frequently in the libraries. These results also indicate that, for libraries with restricted number of heavy atoms to reside mostly within the common drug-like space, the diversity in three-cycle libraries relates more to the combinatorial nature of the library synthetic design (since fewer different BBs are available with acceptable MW), while the chemical diversity of BBs is more important for two-cycle libraries.

A different type of observation that can be extracted directly from the libDESIGN list relates to the frequency in which reactions are utilized in the library designs. This frequency gives a sense of how each reaction couples with the available BBs and provides a measure of the potential utility of that reaction in DEL library preparation. As such, it can guide efforts to further optimize reactions experimentally to maximize their efficiency and highlight reactions and designs which can benefit by the preparation of additional BBs. The frequency of use of BBT incorporation and deprotection reactions in two- and three-cycle designs is available in Supplementary Note 1 and Supplementary Figs. 13–16.

An important conclusion is that the frequency of reaction use does not necessarily match its robustness as perceived by practicing chemists but rather with its complementarity to the available BBs. For example, the triazole synthesis by click chemistry is rarely used due to the lack of available azides as BBs, while amide formation and aldehyde reductive aminations are the most used reactions matching the high abundance of amines, aldehydes, and carboxylic acids in all BB sources.

In order to assess and prioritize libDESIGNs, we conducted an analysis of the selected libraries at the product compound level. For each selected libDESIGN, a random set of compounds referred to as the (X)-Set containing 10,000 compounds was enumerated. The combined (X)-Sets for all the two-cycle and three-cycle designs were used to calculate the average properties of the entire eDESIGNER collection. Library profile and diversity analysis of each specific libDESIGN were calculated with the individual (X)-Sets.

Samples of the enumerated eDESIGNER libraries were compared to two collections of Lilly compounds. The first data set contains random samples of 10,000 compounds from each of the initial 39 DEL libraries synthesized at Lilly before the implementation of eDESIGNER (referred to as the ADEL collection). The second data set is the standard LDC used regularly for screening at Lilly (referred to as the LDC collection). The LDC comprises ~140,000 diverse, drug-like compounds selected as representative of the entire Lilly collection. The samples of each eDESIGNER library were also compared to the cumulative (X)-Set collection in order to identify libraries with unique structural characteristics.

**Fig. 4:**
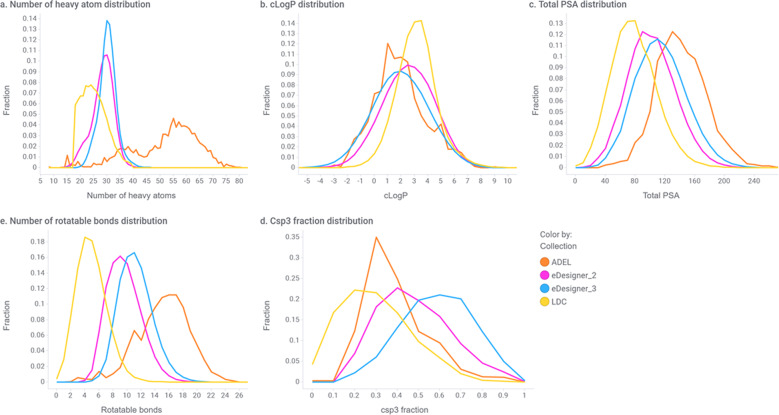
Molecular property profiles. Property distributions for calculated properties. Sample of 10,000 compounds enumerated from each two-cycle libDESIGN (magenta), three-cycle libDESIGN (cyan), previously synthesized libraries (ADEL collection, orange), and 140,000 drug-like compounds belonging to the Lilly diversity cassette (LDC collection, yellow).

Figure 4 and Table 3 summarize the analysis on how the different collections overlap in the chemical property space. The combined (X)-Set for each eDESIGNER library was compared with the ADEL set and the LDC set. As can be observed, the heavy atom distribution limit in eDESIGNER resulted in libDESIGNs with a mean number of heavy atom count within the target unit of 29. It is worth noting that this mean is significantly lower than that of the ADEL set, which was designed with no heavy atom distribution restrictions. Overall, libDESIGN molecules tend to have more rotatable bonds and higher polar surface area than the LDC screening collection but less than the ADEL set. They also tend to have higher Csp3 character, while their lipophilicity is in the low end compared to that of the LDC screening set. Interpretation of these values should consider the headpiece fragment added to all DEL molecules (four atoms, three to four rotatable bonds) not present in molecules in LDC.

**Table 3:** Summary of mean values for selected properties of compounds from the different collections.

Collection	Mean heavy atoms	Mean cLogP	Mean fraction Csp^3^	Mean total polar surface area	Mean number of rotatable bonds
ADEL	51.83	1.92	0.39	141.35	15.09
LDC	25.12	3.14	0.30	78.61	4.94
eDESIGNER_2	28.38	2.53	0.48	102.02	9.59
eDESIGNER_3	30.24	1.97	0.59	114.36	11.23

**Fig. 5:**
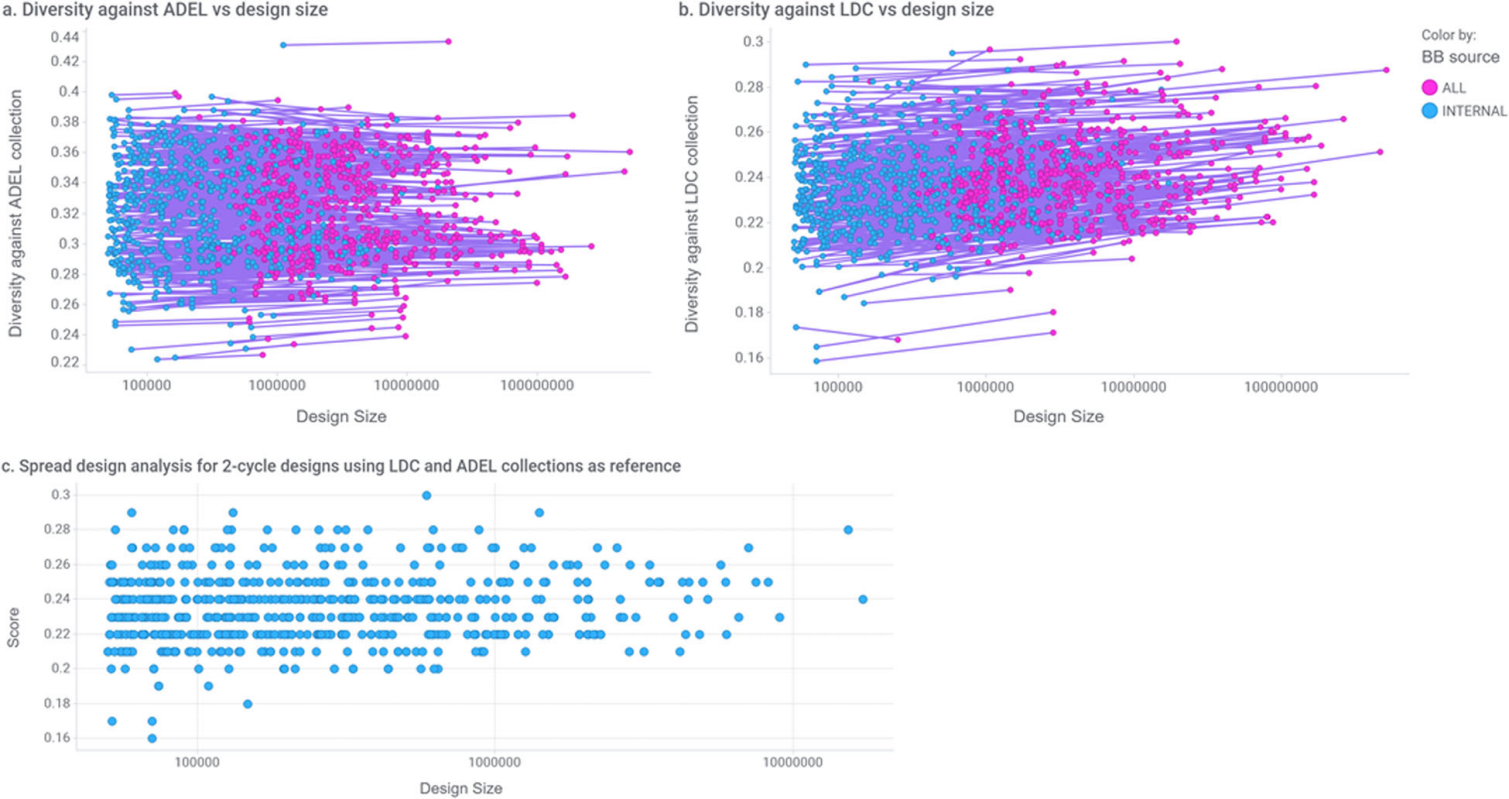
Diversity analysis. **a** Average distance of two-cycle libDESIGN library samples to ADEL. **b** Average distance of two-cycle libDESIGN library samples to LDC collection. Scores are represented versus library size and lines connect enumeration sets (10,000 compounds each) drawn from samples prepared using internal BBs only to samples using all types of BBs. **c** Spread design using the ADEL and LDC collections as reference for two-cycle libDESIGNs using internal BBs only. Scores are represented versus library size.

Figure 5a, b presents our analysis for the two-cycle libraries in terms of distance versus the LDC and ADEL collections. Each dot represents the (X)-Set of a libDESIGN where the *y*-axis is the average distance against the reference collection and the *x*-axis the library size. As can be seen, most of the libraries are sufficiently different from the reference collections with average distances higher than 0.2 calculated using path-based fingerprints and the Tanimoto coefficient (Supplementary Method 3). For each libDESIGN two samples were generated, one using internally available BBs (colored cyan) and the other using both internal and commercial BBs (colored magenta). The dots representing each libDESIGN sample are connected with a line. The length of the line is indicative of the possible library size increase by purchasing BBs from commercial sources; its slope indicates how much additional diversity could be introduced by the vendor BBs. As expected, the overwhelming majority of the lines have a positive slope indicating that the addition of vendor BBs increases the size of the library and adds some diversity. A negative slope is observed in a handful of cases. This is possible when insignificant or no diversity contribution is made by the external BB sets that essentially result in a larger but more homogeneous library set. In this event, the sample set enumerated is also more homogeneous and may contain fewer structurally different chemical structures from the set enumerated using internal BBs.

Panel c presents our spread design analysis on the two-cycle library (X) sets enumerated with internal BBs. Library spread design rank-orders libraries by diversity to a reference set and as such can be used to identify a subset of libraries that is maximally dissimilar to a larger set of libraries. The libraries are ordered from most diverse (highest value on the *y*-axis) to least diverse. In a first step, the average pairwise near neighbor distance of all libDESIGN library samples to the reference sets, in this case the LDC and the Lilly implemented DEL libraries (ADEL), is calculated. The most diverse library, exhibiting a spread value of 0.3, is selected and the reference set is updated with the compounds from that library. The process of distance calculation, selection of most diverse and reference set update, is applied until all libDESIGN libraries have been rank-ordered. In general, library spread design values over 0.25, corresponding to the average near neighbor distance of all library compounds to the original reference set and all libraries already selected, are considered adequate for further consideration. As shown, spread values of two-cycle libDESIGNs with internal BBs range from 0.30 to 0.16 with numerous design choices of varying size in the upper distance range. In a typical scenario, expert chemists review libDESIGNs with higher spread values and accordingly select for experimental implementation. It is worth noting that the diversity analysis described is applied regularly since BB availability continuously changes as experimental library implementation proceeds. Spread design analysis on three-cycle libraries can be found as in Supplementary Note 2.

## Discussion

In summary, eDESIGNER is an algorithm that generates the proximal DEL chemical space by connecting all available BBs with chemical reactions amenable for on-DNA synthesis. The method, already adopted at Lilly as a standard, selects libraries of desired size constrained to a heavy atom distribution more similar to regular screening collections than typical DEL libraries. Finally, the evaluation of virtual compound collections sampled from these designs allows the selection of library designs that add maximum diversity versus previous libraries or available screening cassettes. It is the opinion of the authors that eDESIGNER represents a major improvement in the evaluation of the massive chemical diversity accessible with DEL and a significant step toward the evolution of a rational, data-driven methodology to guide future library synthesis, BB acquisition, and chemical reaction optimization.

## Methods

eDESIGNER implements the different steps in the creation of the libDESIGNS following instructions coded in several parameter files. Explanation and examples of these parameter files are available in Supplementary Method 1: eDESIGNER Parameters.

### BBT creation

The first step in the enumeration of DEL eDESIGNs is to define all possible BBTs and assign available BBs into these BBTs (BBT coding is described in Supplementary Method 2). There are 12,341 possible combinations of three FGs taken from a list of 43 (including the null FG). However, some BBTs can be excluded since certain FG combinations are incompatible. In general, strong electrophiles (e.g., isocyanate and sulphonyl chloride) or strong nucleophiles (e.g., amines and thiols) are considered incompatible among themselves because they would produce non-selectivity issues in many reactions. Moreover, a strong nucleophile is considered incompatible with a strong nucleophile because they could produce BB instability. A detailed explanation of FG incompatibility is available in Supplementary Method 1 and Supplementary Fig. 1. Following elimination of BBTs containing incompatible FGs, the number of possible BBTs is reduced to 6815.

In the next step, BBs from available compound collections are assigned to the correct BBT. The compounds are first “cleaned” by removing fragments corresponding to salts and solvents and standardized via chemical structure canonicalization using RDkit^20^. Common free bases or acids are consolidated in one compound. Compounds are then filtered to eliminate unwanted FGs (the list of unwanted FGs is available in Supplementary Method 1.3) and excessive number of heavy atoms and rotatable bonds. The remaining BBs are classified in one of the 6815 possible BBTs. In order to annotate the BBs with FGs, we use a variation of our previously reported system, in the context of our Proximal Lilly Collection (PLC) technology^21^. eDESIGNER uses an updated version of the PLC annotation incorporating new FGs and hierarchy (e.g., the amine FG comprises both aliphatic and aromatic amines, aliphatic amines comprise both primary and secondary and so on). An example of the command-line tool used for the annotation of BBs is available in Supplementary Method 3. Finally, BBTs without representation are eliminated.

The final number of BBTs depends on both the incompatibilities defined by the user in the parameters provided and the BB collections used. Since chemical collections are dynamic in nature, the number of BBTs could be slightly different in each run. In the study reported here, we obtained 866 BBTs that contained at least one valid BB assigned to them.

### eDESIGNs creation

The second step in the process of creating compound sets for each library design is the enumeration of eDESIGNs. eDESIGNs are graph structures that grow as new reactions and BBTs are added by eDESIGNER. First, the eDESIGN list is initialized by creating one eDESIGN for each possible headpiece (double-stranded DNA fragments that contain a polyethylenglycol linker), each containing a different FG. At each cycle, eDESIGNER attempts a deprotection or scaffold addition followed by a reaction to a new BBT. In order to do that, all possible deprotection reactions are attempted; the ones that match the functionality of the growing eDESIGN generate new eDESIGNs that are added to the list. Each time a reaction is performed, eDESIGNER checks for incompatibilities between reactions and FGs in the growing eDESIGN and incoming BBT. Some incompatibilities with FGs are different when the FG is present in the growing eDESING (on-DNA) or in the incoming BBT (off-DNA). Graphs mapping these incompatibilities are summarized in Supplementary Figs. 1–4. For each growing eDESIGN in the new list all the combinations of reactions and BBTs are attempted and all valid new eDESIGNs generated are kept in a new list of growing eDESIGNs. The process is repeated according to the number of cycles predefined for the final libraries. When all cycles are completed, eDESIGNs that contain FGs not allowed in final molecules are eliminated. A detailed explanation of the eDESIGN creation and codification is available in Supplementary Method 2.5.

### libDESIGNs creation

The reactions used by eDESIGNER are defined at the lowest level of FG type. This means that the same reaction is defined to be different for FGs that have the same parent. For example, an amidation reaction that involves a primary aliphatic amine or a secondary aliphatic amine is defined as different reactions. The two versions of amine FGs are treated distinctly since they can produce different outcomes in some reactions (e.g., a reductive amination). As a result, the number of reactions in eDESIGNER is substantially larger than the number of experimental procedures to perform those reactions. Moreover, different eDESIGNs may be implemented in the same library when they differ only by their use of these equivalent reactions (i.e., an amidation reaction with either primary or secondary amines could be combined in the same library production). This issue is solved by grouping reactions amenable to be combined (both experimentally and for compound enumeration purposes) with a common index (*enum-index*) and combining eDESIGNs that share the same topology and *enum-index* of their reactions into a new graph object called libDESIGN. libDESIGNs are the final objects that define a DEL library in eDESIGNER.

Once the list of libDESIGNs is created, eDESIGNER calculates the largest number of library members achievable while maintaining a predefined heavy atom distribution and eliminates the ones that do not meet the predefined criteria. We selected number of heavy atoms as our main criteria for two considerations: first, the number of heavy atoms can be estimated easily with the distribution of heavy atoms in BBs of each BBT by parameterizing the reactions with the average number of heavy atoms gained or lost in that reaction. This allows us to estimate the heavy atom distribution in a library without enumerating individual compounds, making the algorithm much more efficient. Second, heavy atom distribution, and by extension molecular size, influences other important additive properties such as polar surface area, number of rotatable bonds and, to a lesser extent, lipophilicity, whose distribution is more difficult to estimate without compound enumeration.

A detailed explanation of the libDESIGN creation and coding can be found in Supplementary Method 2. Examples of *enum_reactions* and *enum_deprotections* are available in Supplementary Tables 2 and 3.

### Library enumeration and profiling

Each surviving libDESIGN is translated into a set of instructions recorded in a configuration file that can be processed by our molecule enumeration engine. The eDESIGNER enumeration software has been developed as part of the LillyMol cheminformatics toolkit partially open sourced at https://github.com/EliLillyCo/LillyMol. The software is designed to load a libDESIGN configuration, compile the required BB sets, and run in sequence all steps described therein to virtually synthesize chemical structures. In order to cope with the problem of combinatorial explosion caused by the size of BB sets in multi-step syntheses, the code has been designed to efficiently prepare random samples of any size from the specific libDESIGN space. An example of the command line used to generate samples from a libDESIGN configuration is available in Supplementary Method 3.

In order to determine the sample size to enumerate, we performed a thorough investigation across the entire set of libDESIGNs. Our goal was to identify a sample size small enough to facilitate enumeration and comparison for all libDESIGNs under consideration while sufficiently approximating the property profile of the complete library. To this end, we enumerated random sets of varying sizes ranging between 1000 and 10 million compounds and calculated several molecular properties for each subset to obtain property distributions. Our analysis indicates that subsets of 10,000 compounds and above generate virtually indistinguishable property distributions for the vast majority of libDESIGNs, while subsets of 1000 often present noticeable differences. Based on these observations, we selected 10,000 as a satisfactory compromise of sample size and molecular property distribution approximation for the experiments presented in this paper. Example property distributions plots of varying sample sizes are available in Supplementary Fig. 10 (two cycle) and Supplementary Fig. 11 (three cycle).

The structural diversity of each library can be assessed by calculating and presenting near neighbor distributions of each compound in the library sample to available Lilly compound collections. These distribution plots present an informative visual representation albeit only useful when detailed inspection of a few libraries by a human expert is required. A more comprehensive assessment relies on the calculation of the distance of each libDESIGN data set to the ADEL and LDC reference sets. For the purposes of the experiments presented in this paper, the distance of a pair of libraries (e.g., a specific libDESIGN and the LDC reference set) was calculated by averaging all pairwise tanimoto distances among all the compounds in the two libraries using path-based fingerprints as implemented in the inhouse LillyMol cheminformatics toolkit^22^. Following distance calculation to reference sets, libDESIGNs can be rank-ordered in decreasing order from the most diverse to available Lilly collections, exhibiting a higher value, to the least diverse. Figure 5a, b summarizes the distance values of two-cycle libDESIGNs using BBs from internal and all available sources with respect to library size. An additional method, inspired by algorithms previously proposed to select diverse molecules from large chemical databases^23^, attempts to identify a subset of libraries that is maximally dissimilar from a larger set of libraries. In order to achieve this goal, a measure defining the average distance of libraries is used to generate a pairwise distance matrix of all DEL libraries. The spread design approach^23^ is then applied to rank-order libraries by decreasing adaptive maximal diversity to a reference set. Briefly, the method initially identifies and selects the library with the largest distance to all other libraries and, optionally, any available reference set. The selected library is then combined with the reference set. Subsequent libraries are identified by calculating their distance to the updated reference set, selecting the one with the largest distance and adding it to the reference set. The process terminates when all libraries have been added to the reference set, when a predefined number of libraries have been selected, or when a minimum distance threshold has been reached. Figure 5c plots spread values of two-cycle libDESIGNs using internal BBs over library size using ADEL and LDC as reference sets. The corresponding plot for three-cycle libDESIGNs can be found in Supplementary Method 3 and Fig. 17. The interested reader is referred to Higgs et al.^23^ for a thorough description of spread design methodology. LillyMol command-line examples used to generate fingerprints, calculate near neighbor distances, and calculate the spread of a data set are available in Supplementary Method 3.

## Supplementary information

Supplementary Information

## Data Availability

All data required for the execution of eDESIGNER are available at https://github.com/EliLillyCo/LillyMol and https://github.com/jamflcgh/edesigner_core. External building block data used during this study can be found at corresponding vendor websites. Eli Lilly and Company internal building block data are not available due to IP constraints. All data analysis results generated during this study are included in this published article (and its supplementary information files).

## Code availability

All code used to execute eDESIGNER is available at https://github.com/jamflcgh/edesigner_core (to create the designs) and https://github.com/EliLillyCo/LillyMol (to conduct compound enumeration and property and diversity analysis) and can be used under apache license as specified in each repository.
