# Supplementary material for: Navigating the DNA encoded libraries chemical space
Source: Commun Chem. 2020 Sep 11;3:127. doi: 10.1038/s42004-020-00374-1 (PMC9814293; doi:10.1038/s42004-020-00374-1)
Supplement: Supplementary file 1 — Supplementary Information [file 42004_2020_374_MOESM1_ESM.pdf]

Supplementary information for

Navigating the DNA encoded libraries chemical space

Alfredo Martín*[a], Christos A. Nicolaou*[b], Miguel A. Toledo[a].

a) Eli Lilly and Company, Avda. de la Industria, 30, 28108, Alcobendas, Madrid, Spain, b) Discovery Chemistry, Lilly Research Laboratories, Eli Lilly and Company, Indianapolis, IN 46285. martin_jose_alfredo@lilly.com, c.nicolaou@lilly.com

Table of contents

- Supplementary methods 3
  - 0. eDESIGNER package 3
  - 1. eDESIGNER Parameters 4
    - 1.1.- Functional groups 4
    - 1.2.- Calculated functional groups 4
    - 1.3- Anti-FG 4
    - 1.4.- Reactions 4
    - 1.5.- Enum reactions 5
    - 1.6- Headpieces 5
    - 1.7- par 5
    - 1.8- path 5
  - 2. eDESIGNER Code 22
    - 2.1.- Functional groups (FGs) 22
    - 2.2.- Building Block Types (BBTs) 22
    - 2.3- Incorporation of building blocks to BBTs 22
    - 2.4- Reactions 22
    - 2.5.- Creation of eDESIGNS 23
    - 2.6- Creation of libDESIGNS 24
    - 2.7.- Library compound enumeration 25
  - 3. Sample Commands 31
    - 3.1- Building Block Annotation file preparation example 31
    - 3.2- libDESIGN enumeration example 31
    - 3.3- Fingerprint generation command example 31
    - 3.4- Near neighbor calculation command example 31
- Supplementary Notes 32
  - 1. Reaction utilization 32
  - 2. Spread design analysis 36

Supplementary methods

Supplementary method 0: eDESIGNER package.

eDESIGNER is a package written in python 3 and available at https://github.com/jamflcgh/edesigner_core. It contains 4 main scripts: e_bbt_creator.py, e_designer.py, lib_designer.py and lib_design_interpreter.py

e_bbt_creator reads Building Block Annotation files containing building block collections where each entry contains a smiles string, an identifier for the molecule and a number of functional group (FG) fields. Each functional group entry contains the number of times (count) that the FG is found in the molecule. The *e_bbt_creator* script creates all possible BBTs based on the parameters provided (vide infra), eliminates BBTs bearing incompatible FGs, cleans molecules from the files, assigns molecules to the corresponding BBTs, eliminates BBTs that do not contain any BB and creates a file collection with all BBs grouped by the BBT they belong to. *e_bbt_creator* also creates the BBTs object that will be used by the rest of the scripts.

e_designer creates a list of eDESIGNs using provided parameters (vide infra) and the BBTs object. It outputs a list of eDESIGN objects.

lib_designer creates a list of libDESIGNs using provided parameters and the list of eDESIGNS. It outputs a list of libDESIGNS.

lib_design_interpreter creates a configuration file based on the list of libDESIGNS. The configuration file is used to enumerate samples of compounds from a libDESIGN. Its output is the configuration file that will be described below.

All aforementioned scripts use parameters that regulate their function. The parameters are provided as tab delimited text files with a specific format; the content varies depending on the DNA compatible reaction toolkit available to the user, the functional groups annotated in the collection files by the end user and the specific conditions for the *e_designer* and *lib_designer* runs. In the next sections we describe the parameters used in our organization as an example. The github package contains the parameter files described below as tab delimited text files. It also contains an excel workbook with all the parameters tables to facilitate their edition by the user. File names and paths have been removed from the descriptions due to confidentiality reasons and should be substituted by the ones specified by the user. We will refer to this excel workbook in our explanation of the use of the parameter files.

Supporting LillyMol tools

LillyMol is a cheminformatics toolkit implemented by the Computational Chemistry and Cheminformatics (C3) group at Eli Lill and Company. LillyMol is available at https://github.com/EliLillyCo/LillyMol. The tools tsubstructure and molecular_transformations, iwfp, gfp_nearneighbours, gfp_spread_v2 are used to support the operation of eDesigner.

tsubstructure annotates molecule collections with FG counts and presents the results in an ASCII table format. tsubstructure receives as input a structure file to annotate in smiles format and a file with the functional group queries to use. The tsubstructure tool and the functional group queries used are available as part of LillyMol (see supplementary method 3.1 for an example of use).

molecular_transformations enumerates structures using library design configuration files that define reaction and building block information. The molecular_transformations tool and the reactions used are available as part of LillyMol (see Supplementary method 3.2 for an example of use and Supplementary method 2.7 for a description of library configuration file format).

iwfp calculates path-based fingerprints (see Supplementary method 3.3 for an example of use)

gfp_nearneighbours calculates near neighbor distances of 2 sets of compound fingerprints (see Supplementary method 3.4 for an example of use)

gfp_spread_v2 calculates the spread distance of compounds in a set to rank-order by diversity (see Supplementary method 3.5 for an example of use)

Supplementary method 1: eDESIGNER Parameters.

1.1.- Functional groups are provided in a table such as the one exemplified in spreadsheet ***fg*** in the parameters workbook. The column *index* indicates the index for the functional group (consecutive integer) starting at 0 with the null FG (used as placeholder for a non-existing functional group).

name indicates the given name of the FG as in the BB file. Some of the names of the FGs start with the word "calc". This is due to the hierarchical nature of the functional groups as annotated in our chemical databases. For example, the NITRO_FLUORO FG (15) is defined as an aryl group with a fluorine atom ortho to a nitro group. This FG is more elaborated than a regular mono-dentate group such as a primary aliphatic amine or an aldehyde, and is useful to construct, for example, the benzimidazole scaffold by the reaction sequence composed by a nucleophilic aromatic substitution followed by reduction of the nitro group in the presence of an aldehyde. All building blocks containing this FG will have the o-fluoro nitroarene substructure, which will be counted as an instance of FG of index 15. However, these building blocks also contain the nitro substructure and therefore the NITRO FG instance will be counted as well. To avoid double counting of the same substructures in a building block as different FGs (which would make eDESIGNER create incorrect designs) we have modified the counting of the FG at the higher hierarchical level, in this case the NITRO FG, by subtracting the instances of the o-fluoro nitroarene substructure from the instances of the nitro substructure. The FG resulting from this operation is labeled calc_NITRO functional group (8). The end result of this operation is that the o-fluoro nitroarene will be excluded to participate in other reactions that use exclusively the Nitro group. This is acceptable since the number of building blocks containing o-fluoro nitroarenes is less than the the number of molecules containing the nitro FG. The calculation of all the "calculated functional groups" is summarized in spreadsheet ***calcfg*** in the parameters workbook. Chemical drawings exemplifying the functional groups are presented in table 1.

The electrophiles for the nucleophilic aromatic substitution (calc_NAS_ELECTROPHILE, 37) are an especially difficult case, not only because it lays on the top hierarchy of other electrophiles, but also because it cannot be easily defined as a simple substructures search. This functional group was defined in turn with multiple substructure searches comprising a leaving group in an aromatic core and one or more activating electrophiles.

Each functional group is characterized and coded for other characteristics including whether or not the functional group is stable when present in a molecule attached to DNA (column: *stable*); how many atoms (column *atom_dif*) or how many rotatable bonds (column: *excess_rb*) the functional group loses on average upon its participation in a typical reaction; and whether library member molecules that carry this specific functional group at the end of the library synthesis will be allowed or not (column: *allowed_end_exposed*)

The *self_incompatibility* column indicates the indexes of functional groups that are incompatible to coexist with the current one in a molecule. Typically, strong electrophiles are incompatible with strong nucleophiles; moreover, they are usually incompatible among themselves since coexistence could drive to lack of selectivity in the reactions. To address this behavior, all functional groups are incompatible with a copy of themselves except the null FG. The current incompatibility column was prepared based on our experience. Users can modify it to get different results as per their specific needs.

Supplementary figure 1 depicts graphically the incompatibilities of the functional groups as in our definition.

1.2.- Calculated functional groups: As mentioned above some functional groups are calculated on the fly by e_bbt_creactor. The rules for these calculations are collected in the spreadsheet ***calcfg*** in the parameters workbook, which is supplied to e_bbt_creator as a parameters file.

name column indicates the final name for the functional group. *rule_add* and *rule_substract* are lists (';' separated) of FG names as they appear in the BB file. *e_bbt_creator* adds the number of instances that the FGs in the *rule_add* list appear in the BB file for a specific BB and subtracts the number of instances that the FGs in the *rule_substract* list appear in the BB file for that specific BB to compute the number of instances for the calculated FG.

1.3- Anti-FG: *e_bbt_creator* eliminates all building blocks containing not desired functional groups. The list of these functional groups is provided as the Anti-FG parameters table (***antifg*** in the parameters workbook). Some of these functional groups are actually calc_FGs so they are calculated on the fly as described above.

1.4.- Reactions are provided as parameters tables (note that reactions are parametrized, in this manuscript we have used reaction in Lilly's toolkit but different reactions could be coded giving rise to different designs). Spreadsheet ***reaction*** in the parameters workbook contains the connecting reactions table and spreadsheet ***deprotection*** contains the deprotection/scaffold incorporation reactions. The format is the same in both. The first column is the *index* of the reaction. The second is the *fg_input_on_off* field. This is a list of two integers (';' separated) that contain the indexes of the FGs that participate in the reaction: the one coming from the growing eDESIGN (on DNA) and the one coming from the incoming BBT (off DNA). In the case of deprotection reactions the second number is always 0 since there is no incoming BBT. The *fg_out_on_off* indicates a pair of functional groups that are generated upon reaction. If no reactive functional group is created both numbers are 0; if only one FG is created it is indicated in the first position of the tuple; if two FGs are created the two members of the tuple become non-zero. The functional groups created become members of the growing eDESIGN (they are positioned on DNA for future reactions).

The column *excluded_on* is a list of FGs that are incompatible with this reaction when they are on DNA (they come with the growing eDESIGN). The *excluded_off* is the list of FGs that are incompatible with the reaction when they come with the incoming BBT. As with the incompatibility rules in the FGs, the reaction incompatibilities are derived from our experience, but can be modified by users. Tip: when attempting such modification the first number in the *fg_input_on_off* cannot be included in the *excluded_on* list and the second number cannot be included in the *excluded_off* list, otherwise the reaction would never happen. Supplementary figures 2, 3 and 4 are graphical depictions of the incompatibility matrices for enumeration and BBT incorporation reactions

The *name* field indicates the given name of the reaction. We have defined the name format as a number indicating the hierarchy in the RXNO reaction ontology, followed by the reaction common name, the name of the FG used coming from the growing eDESIGN and the name of the FG from the incoming BBT. For example:

"1.2.1_ALDEHYDE_REDUCTIVE_AMINATION_FROM_ALDEHYDES_AND_AMINES_ALIPHATIC_PRIMARY".

The *production* field indicates whether this reaction is in production or in development. The *atom_dif* field indicates the number of heavy atoms that are gained by the design because of this reaction, excluding the ones coming from the BB. The *end_deprotect* field for the deprotection reactions indicates whether to conduct this deprotection after the last cycle and the *enum_index* indicates the index of the enumeration reaction corresponding to this reaction

1.5.- Enum reactions are provided in the spreadsheets ***enum_reaction*** and ***enum_deprotection*** in the parameters workbook. The enum_reactions are reaction groups that comprise several reactions that can be conducted in the same experimental conditions. Each table contains an i*ndex* (which is referred to in the reaction parameters tables) and a given name of the reaction under the *enum_name* field. The *enum_reactions*, and not the *reactions* described in the previous method, are the ones coded to enumerate samples for the libDESIGNS. Their codification can be found in **LillyMol.** Tables 2 and 3 depict an example for each *enum_reaction* and *enum_deprotection* respectively.

1.6- Headpieces are attachment points to the double stranded DNA that are used to grow the molecules in the DEL libraries. The attachment point is a functional group, and for computation purposes, it is assigned to a BBT. Headpiece parameters table is provided in the spreadsheet ***headpieces*** in the parameters workbook. The table contains the following fields the following fields: *index* is an integer number that identifies the headpiece. *bbt* is a list of three integers representing the FGs in the headpiece. This tuple of integers represents the BBT the headpiece belongs to. *fg* is a list of the names of the FGs in the BBT and *smiles* is the smiles string that will be used in the enumeration of final molecules representing the headpiece. It always contains a ^{13}C atom at the opposite end of the FG used to grow the molecule to easily identify the headpiece in the molecule.

1.7- par parameters (spreadsheet ***par*** in the parameters workbook) are the main parameters that guide *e_designer* and *lib_designer* how to create designs. The *max_na_percentile* field indicates the maximum number of heavy atoms in the percentile of the distribution indicated in the field *percentile*. In the example, *percentile* is set to 0.5 so 29 atoms will correspond to the median of the distribution. *max_na_absolute* is the maximum number of atoms allowed for a molecule in the design. *max_cycle_na* is a list (';' separated) with the maximum number of atoms for the smallest molecule allowed at each cycle. This field indicates also how many cycles the designs will have (the number of members in this list). The *max_scaffold_na* field indicates the maximum number of heavy atoms that can be incorporated as scaffolds. *headpiece_na* is the number of atoms coming from the headpiece. *min_count* is the minimum library size coming from a lib_design, just considering the BBs coming from internal sources, for the designs to be accepted. The field *include_designs* indicates the type of reactions that will be used to create designs (PRODUCTION for production reactions or BOTH for both production and development reactions). The field *rb_filter* indicates the maximum number of effective rotatable bonds allowed for a BB to be considered. The field *designs_in_memory* indicates the maximum number of designs held in memory while expanding a list of designs. *e_designer* will save to disk the list of designs generated at a given cycle and will read to memory these designs in lists of the size indicated by this parameter. This is done to avoid memory overflow. The user should use the appropriate value depending on hardware memory capacity. The fields *final_compounds_folder* and *final_reactions_folder* are the paths indicating the folder containing the reaction files used for enumeration and the folder where design level combined BB files is stored. These values will go unchanged to the configuration file.

1.8- path parameters (spreadsheet ***path*** in the parameters workbook) control where the different files and logs are stored and the names of the files corresponding to logs and results. *Database_Run* is a token that is generated by *e_bbt_creator*, using the date of the e_bbt_creator run. This is the date where compound collection files are processed into BB containing BBTs files (collection files are updated daily at Lilly). A folder with the name of this token is generated under "comps" folder and all the files for the BBs organized by BBT are placed in this folder. In order to instruct eDESIGNER to use the appropriate BB set this token must be provided as a parameter (*Database_Run*). This token is also used as a prefix of all files generated in subsequent runs by *e_designer*, *lib_designer* and *lib_design_interpreter* when using the BBs generated this date. Since different run conditions can be performed by *e_designer* and *lib_designer* with the same version of BBs, an id for the specific run is provided as parameter *run*. This value is used as a second prefix for files created by *e_designer*, *lib_designer* and *edesign_interpreter.*

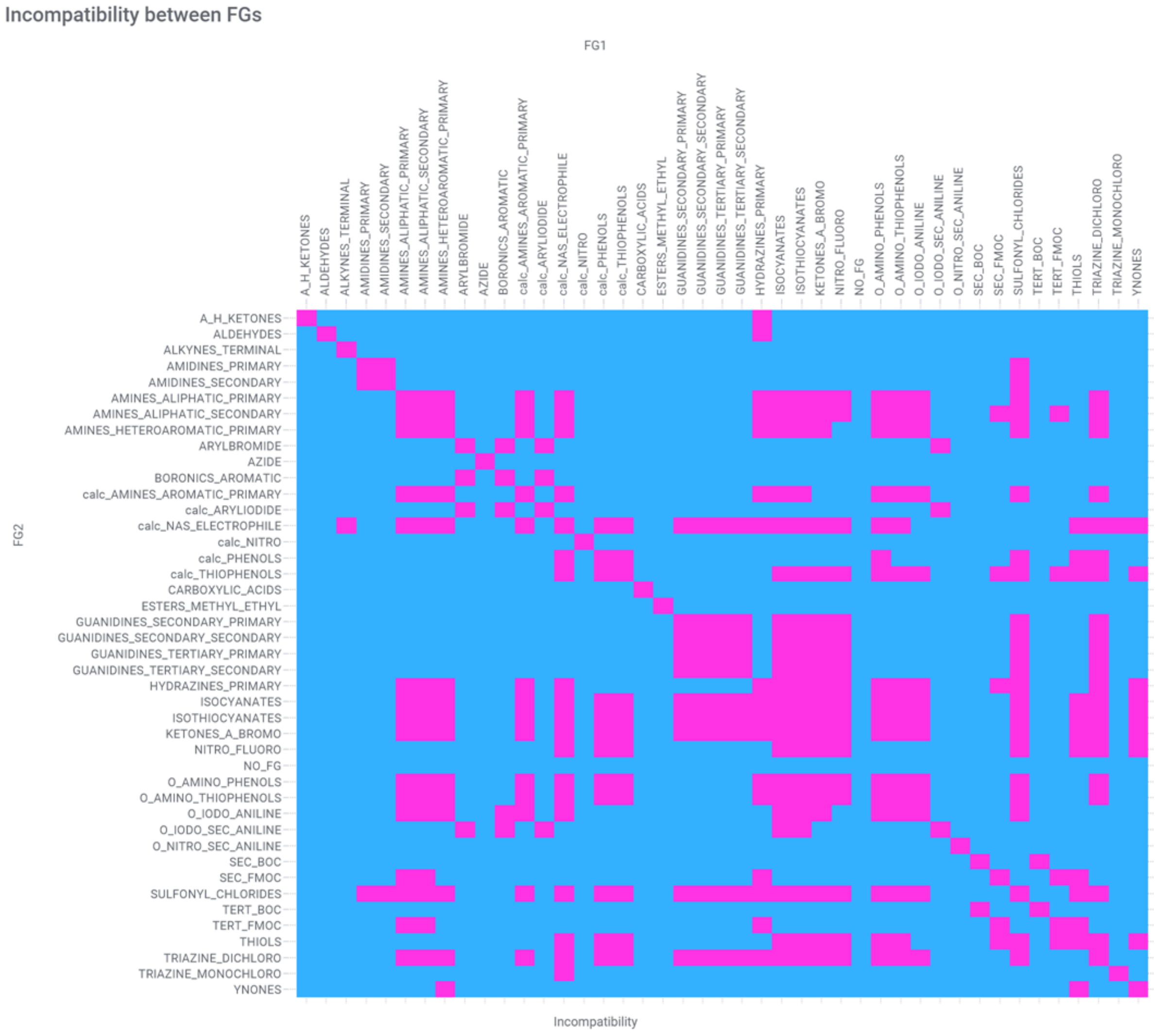

Supplementary Figure 1: Incompatibility matrix for functional groups in the same BBT. Magenta color means incompatible, cyan color means compatible.

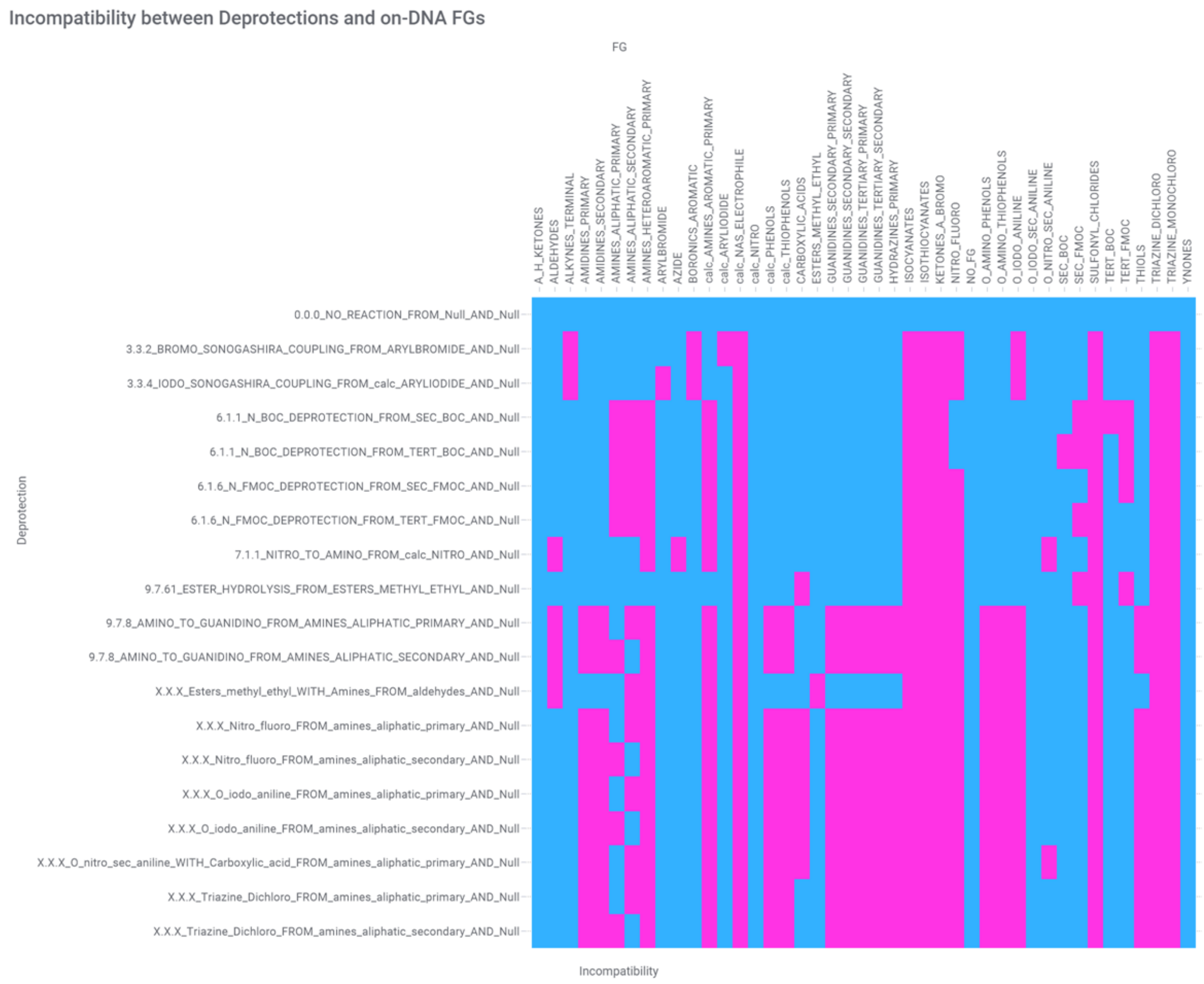

Supplementary Figure 2: Incompatibility matrix for functional groups regarding deprotection reactions. Magenta color means incompatible, cyan color means compatible

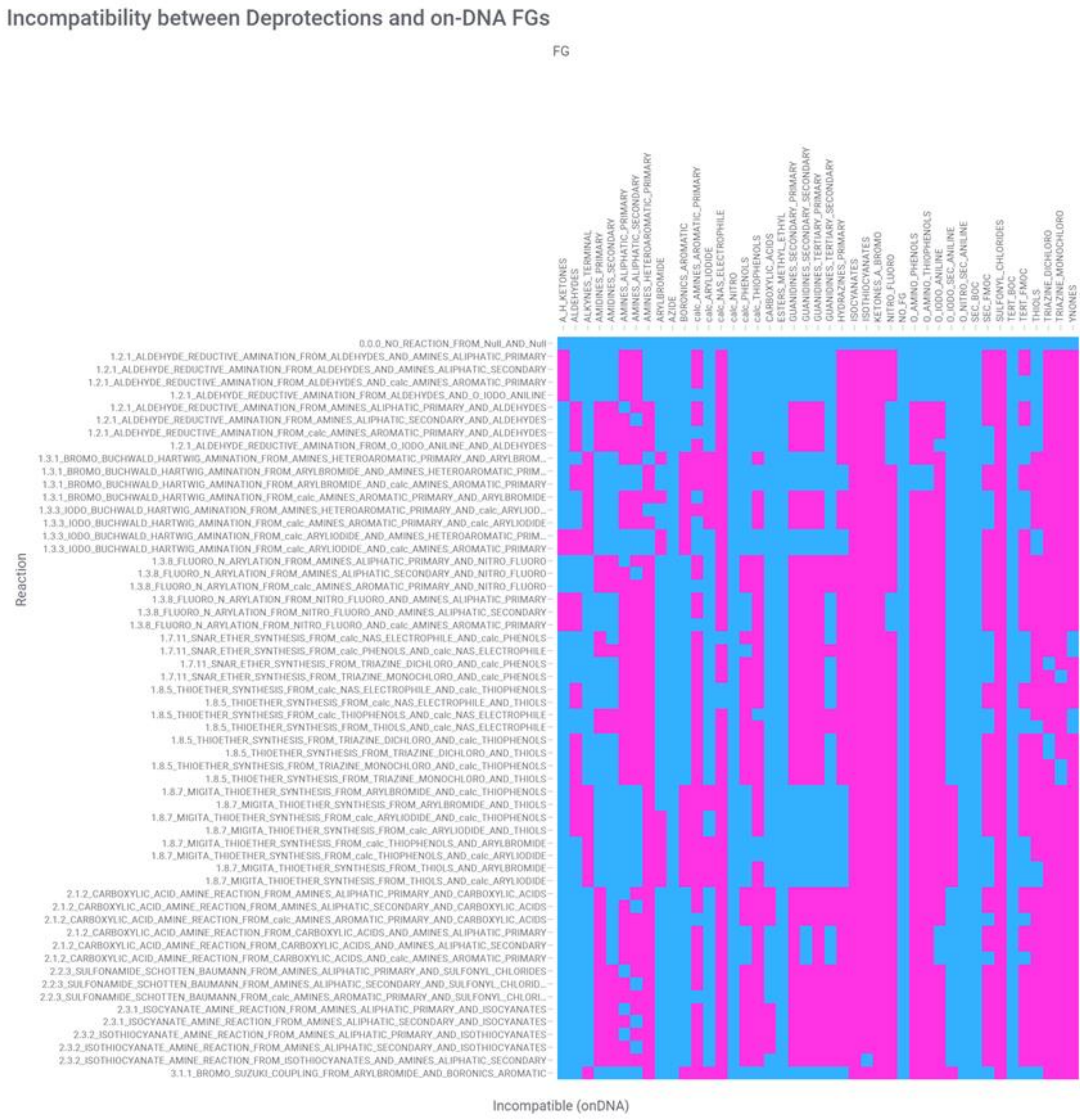

Supplementary Figure 3 (1): Incompatibility matrix for functional groups *on DNA* regarding reactions. Magenta color means incompatible, cyan color means compatible

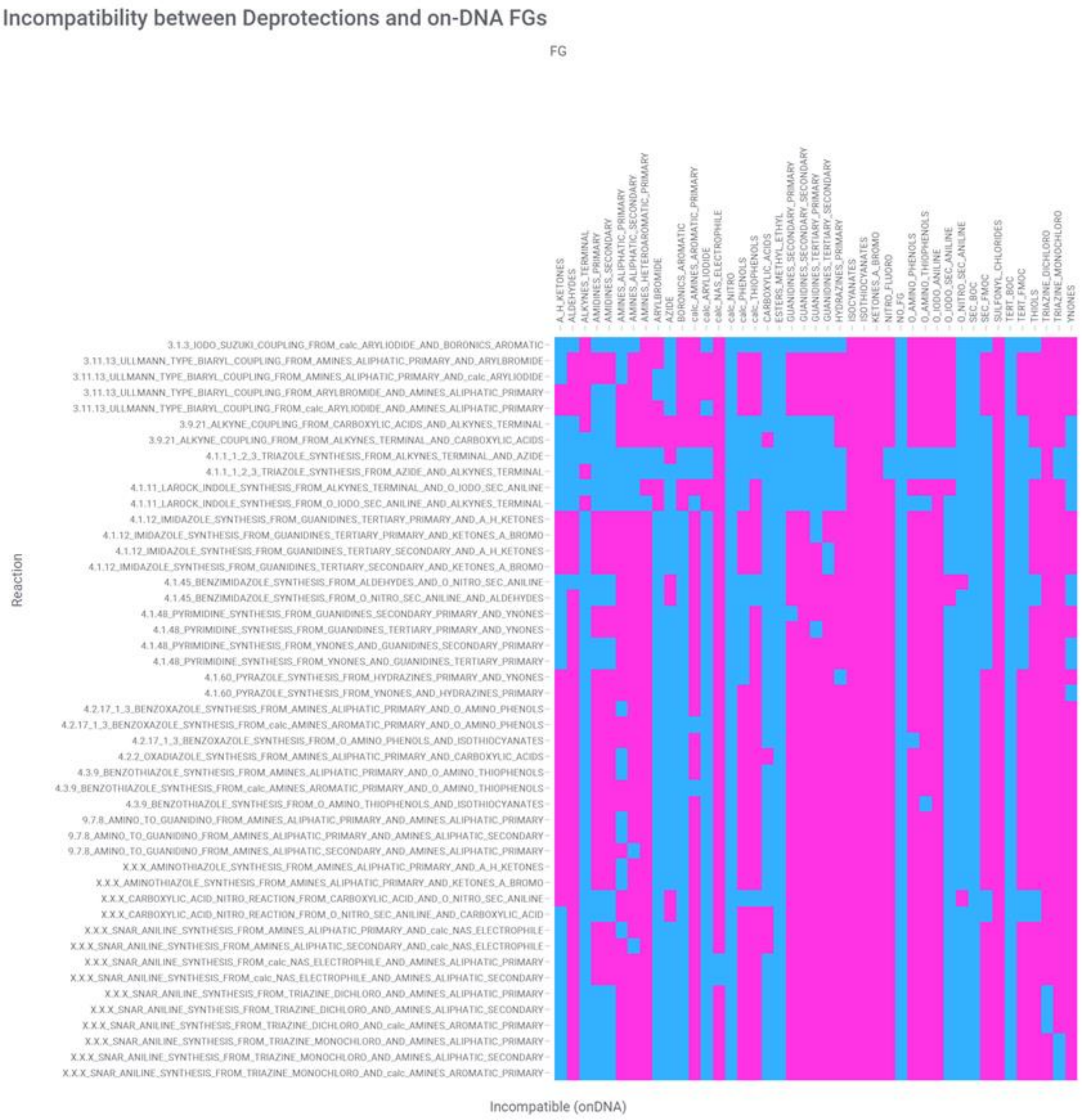

Supplementary Figure 3 (2): Incompatibility matrix for functional groups *on DNA* regarding reactions. Magenta color means incompatible, cyan color means compatible

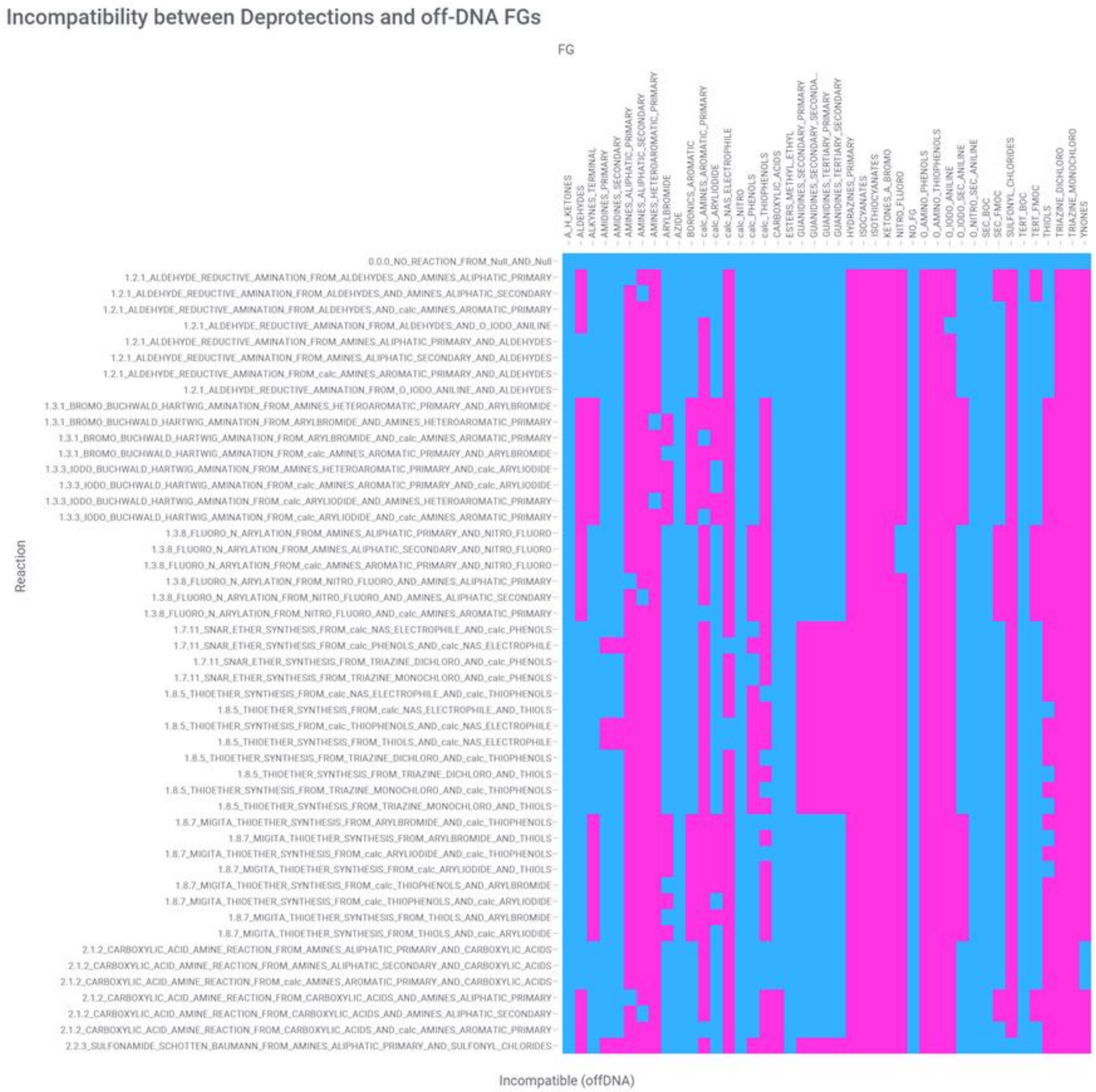

Supplementary Figure 4 (1): Incompatibility matrix for functional groups *off DNA* regarding reactions. Magenta color means incompatible, cyan color means compatible

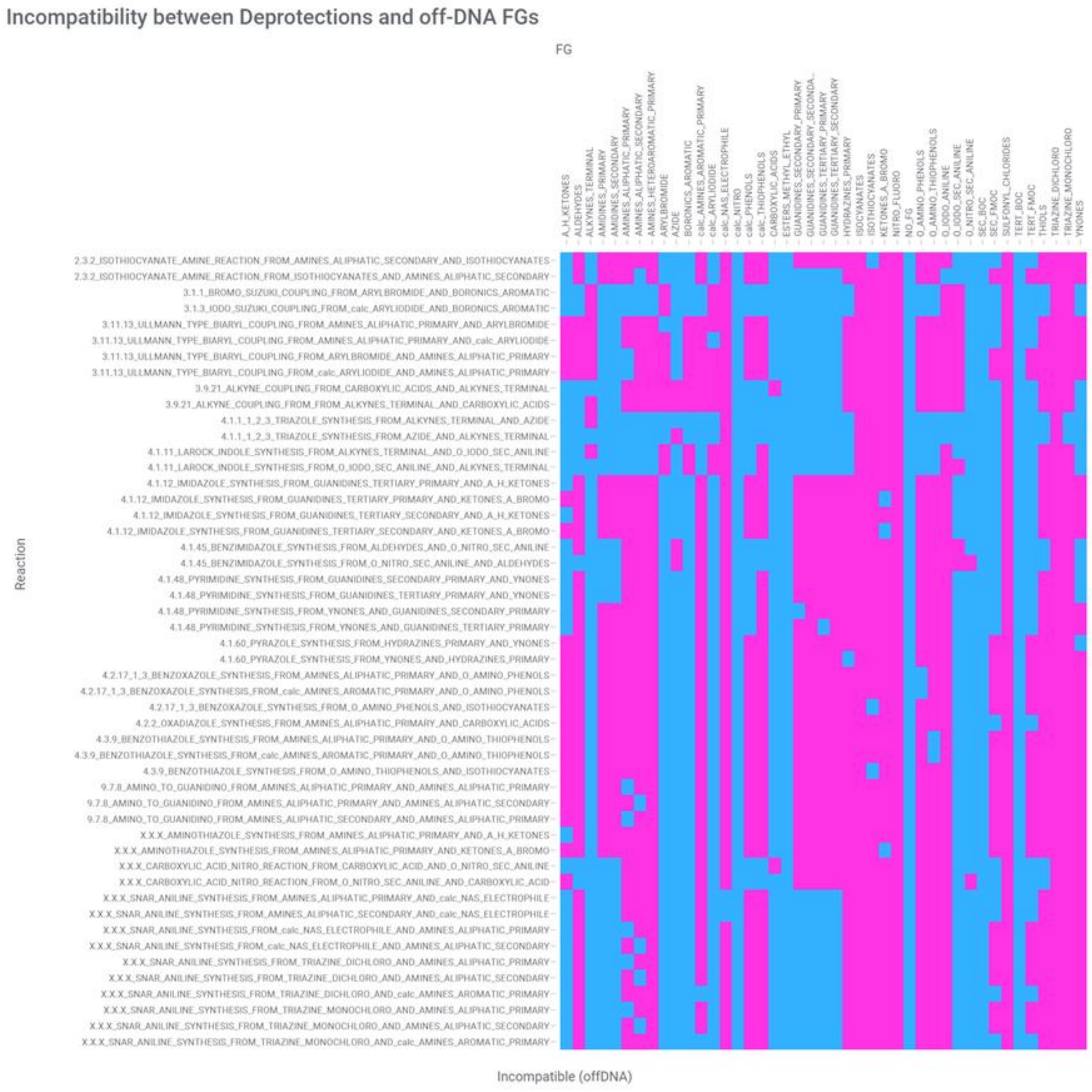

Supplementary Figure 4 (2): Incompatibility matrix for functional groups *off DNA* regarding reactions. Magenta color means incompatible, cyan color means compatible

Supplementary Table 1: Figures of functional group examples

id	Name	Example
1	ALDEHYDES	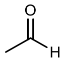
2	ALKYNES_TERMINAL	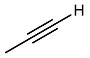
3	AMINES_ALIPHATIC_SECONDARY	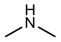
4	AZIDE	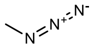
5	CARBOXYLIC_ACIDS	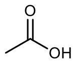
6	THIOLS	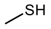
7	calc_THIOPHENOLS	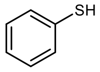
8	calc_NITRO	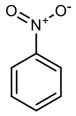
9	calc_PHENOLS	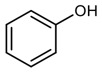
10	SEC_FMOC	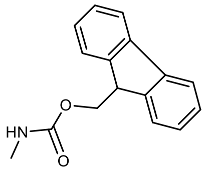
11	SULFONYL_CHLORIDES	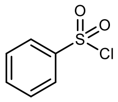
12	BORONICS_AROMATIC	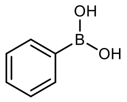
13	TERT_FMOC	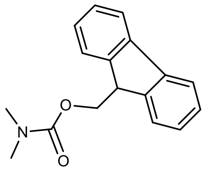
14	ESTERS_METHYL_ETHYL	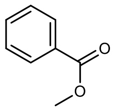
15	NITRO_FLUORO	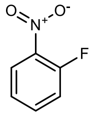
16	SEC_BOC	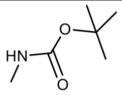
17	TERT_BOC	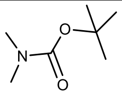
18	O_NITRO_SEC_ANILINE	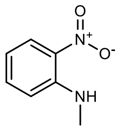

19	calc_ARYLIODIDE	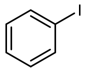
20	ARYLBROMIDE	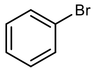
21	AMINES_ALIPHATIC_PRIMARY	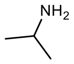
22	calc_AMINES_AROMATIC_PRIMARY	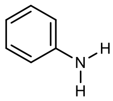
23	AMINES_HETEROAROMATIC_PRIMARY	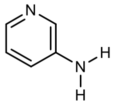
24	ISOCYANATES	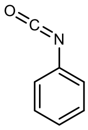
25	ISOTHIOCYANATES	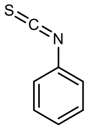
26	O_IODO_ANILINE	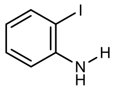
27	HYDRAZINES_PRIMARY	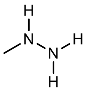
28	A_H_KETONES	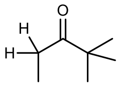
29	KETONES_A_BROMO	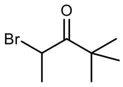
30	YNONES	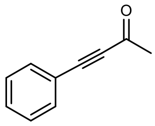
31	AMIDINES_PRIMARY	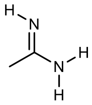
32	AMIDINES_SECONDARY	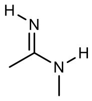
33	GUANIDINES_SECONDARY_PRIMARY	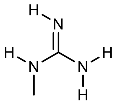
34	GUANIDINES_SECONDARY_SECONDARY	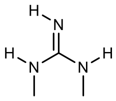
35	GUANIDINES_TERTIARY_PRIMARY	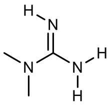
36	GUANIDINES_TERTIARY_SECONDARY	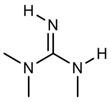

37	calc_NAS_ELECTROPHILE	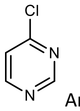 Among several other substructures
38	O_AMINO_PHENOLS	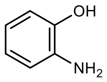
39	O_AMINO_THIOPHENOLS	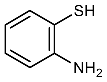
40	O_IODO_SEC_ANILINE	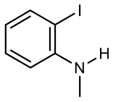
41	TRIAZINE_DICHLORO	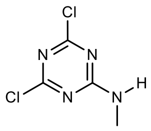
42	TRIAZINE_MONOCHLORO	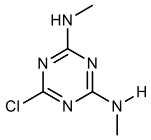

Supplementary Table 2: Figures of eDESIGNER coupling reactions examples

id	Name	Example
1	1.2.1_Aldehyde_reductive_amination_FROM_aldehydes_AND_amines.rxn	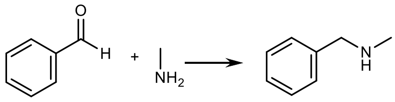
2	1.2.1_Aldehyde_reductive_amination_FROM_amines_AND_aldehydes.rxn	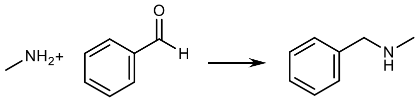
3	1.3.1_Bromo_Buchwald-Hartwig_amination_FROM_arylbromide_AND_amines_aromatic.rxn	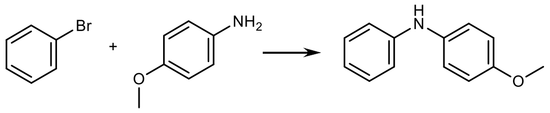
	1.3.1_Bromo_Buchwald-Hartwig_amination_FROM_amines_aromatic_AND_arylbromide.rxn	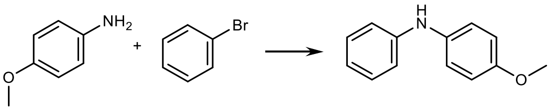
	1.3.3_Iodo_Buchwald-Hartwig_amination_FROM_amines_aromatic_AND_aryliodide.rxn	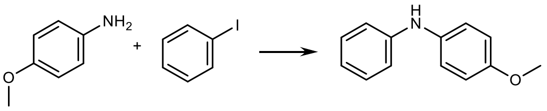
	1.3.3_Iodo_Buchwald-Hartwig_amination_FROM_aryliodide_AND_amines_aromatic.rxn	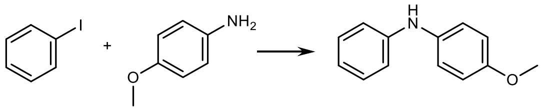
	1.3.8_Fluoro_N-arylation_FROM_amines_AND_nitro_fluoro.rxn	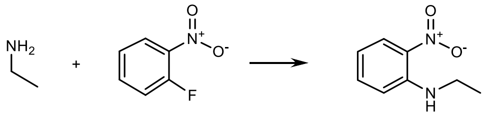
	1.3.8_Fluoro_N-arylation_FROM_nitro_fluoro_AND_amines.rxn	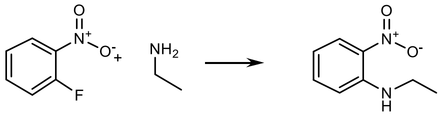
	1.7.11_SNAr_ether_synthesis_FROM_phenols_AND_NAS_electrophile.rxn	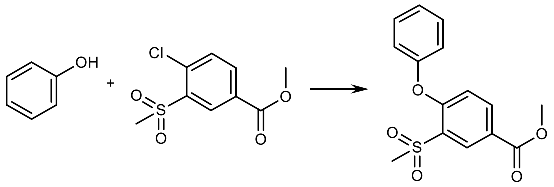
	1.7.11_SNAr_ether_synthesis_FROM_NAS_electrophile_AND_phenols.rxn	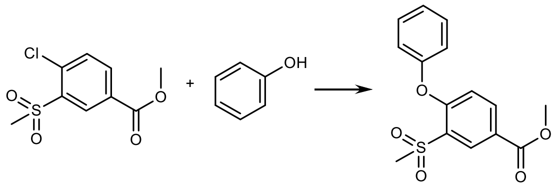
	1.7.11_SNAr_ether_synthesis_FROM_triazine_chloro_AND_phenols.rxn	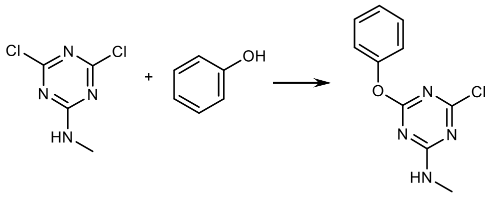
	1.8.5_Thioether_synthesis_FROM_thiophenols_AND_NAS_electrophile.rxn	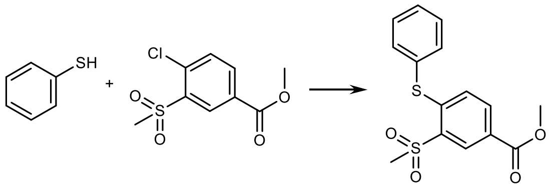

	1.8.5_Thioether_synthesis_FROM_NAS_electrophile_AND_thiophenols.rxn	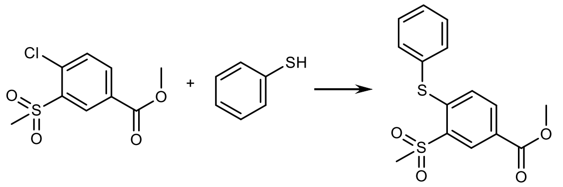
	1.8.5_Thioether_synthesis_FROM_NAS_electrophile_AND_thiols.rxn	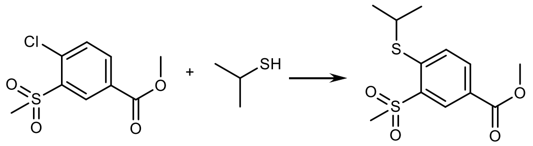
	1.8.5_Thioether_synthesis_FROM_thiols_AND_NAS_electrophile.rxn	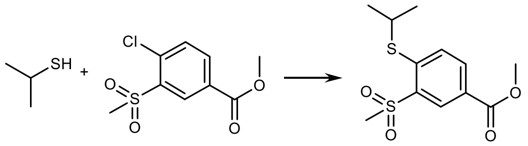
	1.8.5_Thioether_synthesis_FROM_triazine_chloro_AND_thiophenols.rxn	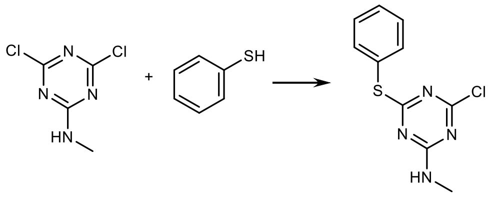
	1.8.5_Thioether_synthesis_FROM_triazine_chloro_AND_thiols.rxn	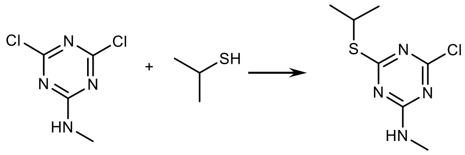
	1.8.7_Migita_thioether_synthesis_FROM_arylbromide_AND_thiophenols.rxn	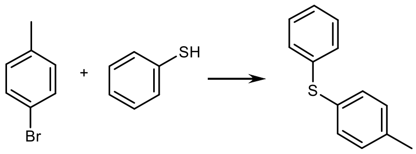
	1.8.7_Migita_thioether_synthesis_FROM_arylbromide_AND_thiols.rxn	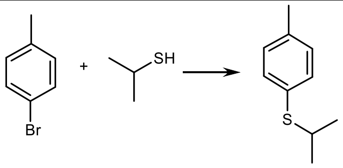
	1.8.7_Migita_thioether_synthesis_FROM_aryliodide_AND_thiophenols.rxn	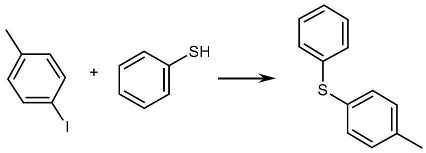
	1.8.7_Migita_thioether_synthesis_FROM_aryliodide_AND_thiols.rxn	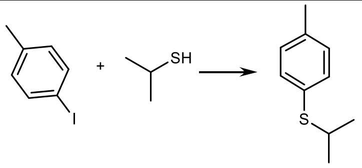
	1.8.7_Migita_thioether_synthesis_FROM_thiophenols_AND_arylbromide.rxn	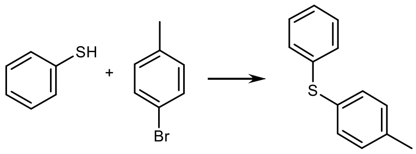
	1.8.7_Migita_thioether_synthesis_FROM_thiophenols_AND_aryliodide.rxn	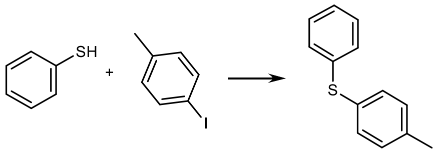

	1.8.7_Migita_thioether_synthesis_FROM_thiols_AND_arylbromide.rxn	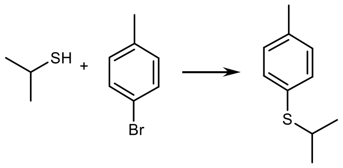
	1.8.7_Migita_thioether_synthesis_FROM_thiols_AND_aryliodide.rxn	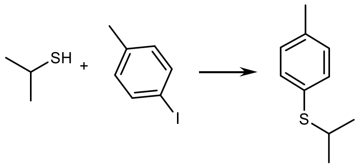
	2.1.2_Carboxylic_acid_+_amine_condensation_FROM_amines_AND_carboxylic_acids.rxn	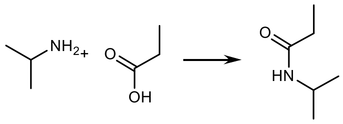
	2.1.2_Carboxylic_acid_+_amine_condensation_FROM_carboxylic_acids_AND_amines.rxn	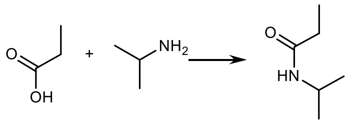
	2.2.3_Sulfonamide_Schotten-Baumann_FROM_amines_AND_sulfonyl_chlorides.rxn	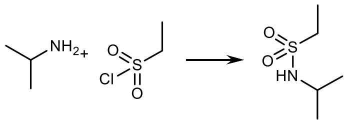
	2.3.1_Isocyanate_+_amine_urea_coupling_FROM_amines_aliphatic_AND_isocyanates.rxn	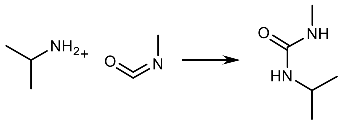
	2.3.2_Isothiocyanate_+_amine_thiourea_coupling_FROM_amines_aliphatic_AND_isothiocyanates.rxn	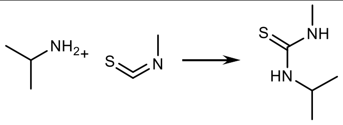
	2.3.2_Isothiocyanate_+_amine_thiourea_coupling_FROM_isothiocyanates_AND_amines_aliphatic.rxn	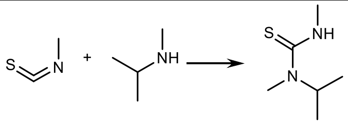
	3.1.1_Bromo_Suzuki_coupling_FROM_arylbromide_AND_boronics.rxn	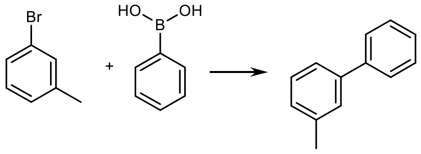
	3.1.3_Iodo_Suzuki_coupling_FROM_aryliodide_AND_boronics.rxn	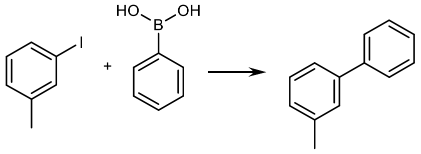
	3.11.13_Ullmann-type_biaryl_coupling_FROM_amines_aliphatic_primary_AND_arylbromide.rxn	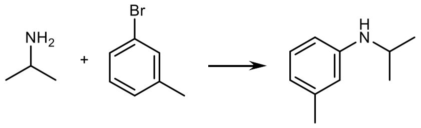
	3.11.13_Ullmann-type_biaryl_coupling_FROM_amines_aliphatic_primary_AND_aryliodide.rxn	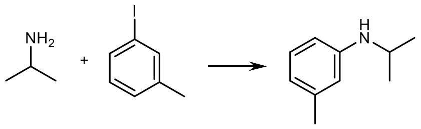
	3.11.13_Ullmann-type_biaryl_coupling_FROM_arylbromide_AND_amines_aliphatic_primary.rxn	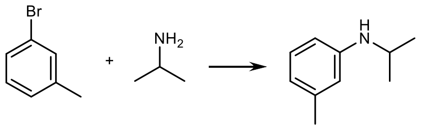

	3.11.13_Ullmann-type_biaryl_coupling_FROM_aryliodide_AND_amines_aliphatic_primary.rxn	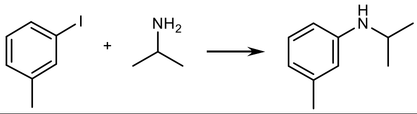
	3.9.21_Alkyne_coupling_FROM_alkynes_terminal_AND_carboxylic_acids.rxn	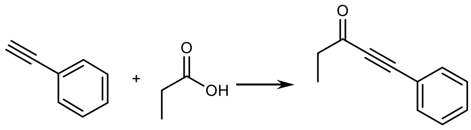
	3.9.21_Alkyne_coupling_FROM_carboxylic_acids_AND_alkynes_terminal.rxn	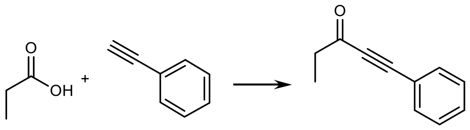
	4.1.1_1_2_3-Triazole_synthesis_FROM_azide_AND_alkynes_terminal.rxn	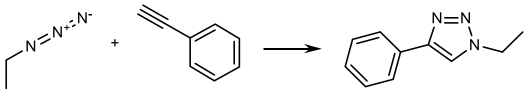
	4.1.1_1_2_3-Triazole_synthesis_FROM_alkynes_terminal_AND_azide.rxn	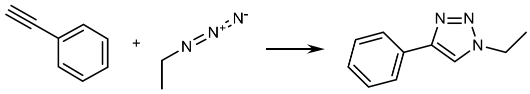
	4.1.11_Larock_indole_synthesis_FROM_alkynes_terminal_AND_o_iodo_aniline.rxn	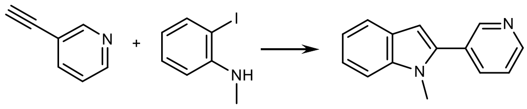
	4.1.11_Larock_indole_synthesis_FROM_o_iodo_aniline_AND_alkynes_terminal.rxn	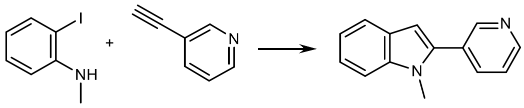
	4.1.12_Imidazole_synthesis_FROM_guanidines_tertiary_primary_AND_ketones_a_bromo.rxn	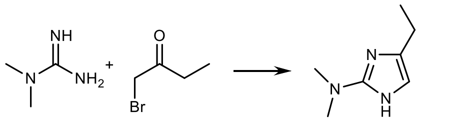
	4.1.12_Imidazole_synthesis_FROM_guanidines_tertiary_primary_AND_a_h_ketones.rxn	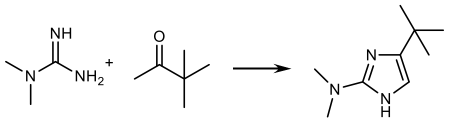
	4.1.12_Imidazole_synthesis_FROM_guanidines_tertiary_secondary_AND_ketones_a_bromo.rxn	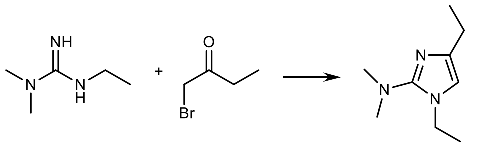
	4.1.12_Imidazole_synthesis_FROM_guanidines_tertiary_secondary_AND_a_h_ketones.rxn	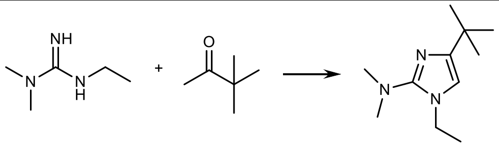
	4.1.45_Benzimidazole_synthesis_FROM_aldehydes_AND_o_nitro_sec_aniline.rxn	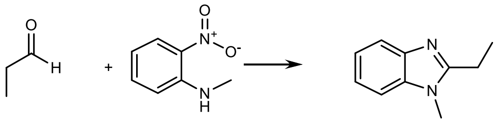
	4.1.45_Benzimidazole_synthesis_FROM_o_nitro_sec_aniline_AND_aldehydes.rxn	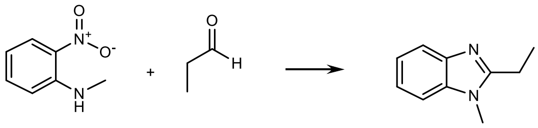
	4.1.48_Pyrimidine_synthesis_FROM_guanidines_sectert_primary_AND_ynones.rxn	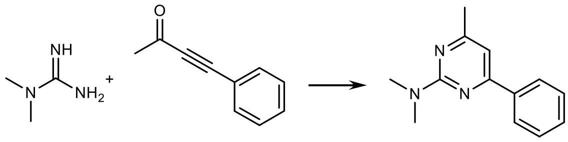

	4.1.48_Pyrimidine_synthesis_FROM_ynones_AND_guanidines_sectert_primary.rxn	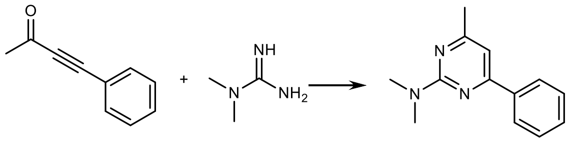
	4.1.60_Pyrazole_synthesis_FROM_ynones_AND_hydrazines_primary.rxn	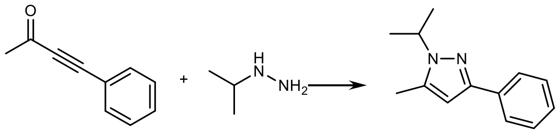
	4.1.60_Pyrazole_synthesis_FROM_hydrazines_primary_AND_ynones.rxn	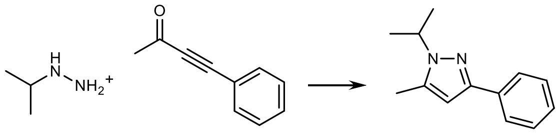
	4.2.17_1,3-Benzoxazole_synthesis_FROM_amines_aliphatic_primary_AND_o_amino_phenols.rxn	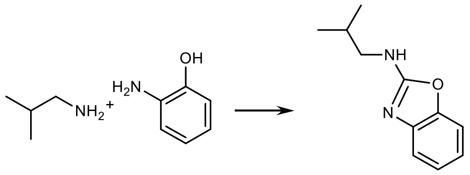
	4.2.17_1,3-Benzoxazole_synthesis_FROM_amines_aromatic_primary_AND_o_amino_phenols.rxn	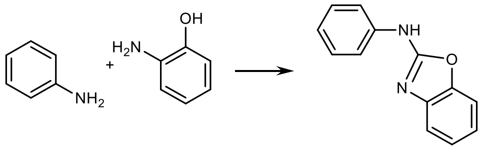
	4.2.17_1,3-Benzoxazole_synthesis_FROM_o_amino_phenols_AND_isothiocyanates.rxn	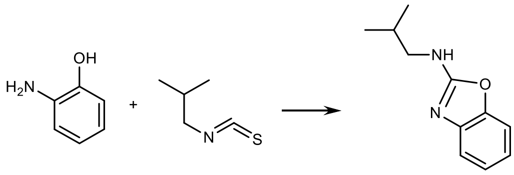
	4.2.2_1,2,4-Oxadiazole_synthesis_FROM_amines_aliphatic_primary_AND_carboxylic_acids.rxn	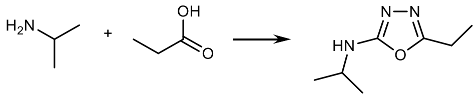
	4.3.9_Benzothiazole_synthesis_FROM_amines_aliphatic_primary_AND_o_amino_thiophenols.rxn	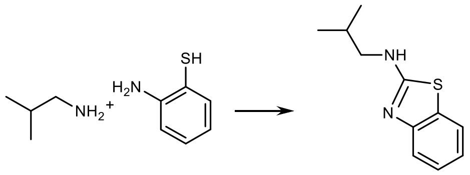
	4.3.9_Benzothiazole_synthesis_FROM_amines_aromatic_primary_AND_o_amino_thiophenols.rxn	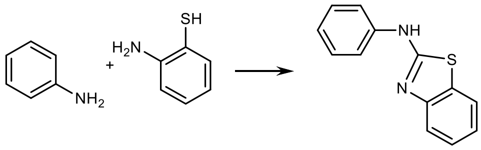
	4.3.9_Benzothiazole_synthesis_FROM_o_amino_thiophenols_AND_isothiocyanates.rxn	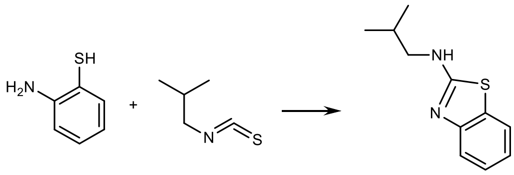
	9.7.8_Amino_to_guanidino_FROM_amines_aliphatic_AND_amines_aliphatic.rxn	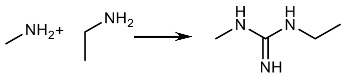
	X.X.X_Aminothiazole_synthesis_FROM_amines_aliphatic_primary_AND_ketones_a_bromo.rxn	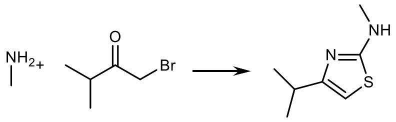

	X.X.X_Aminothiazole_synthesis_FROM_amines_aliphatic_primary_AND_a_h_ketones.rxn	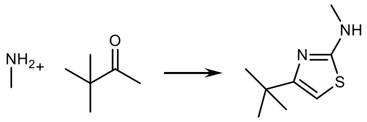
	X.X.X_SNAr_aniline_synthesis_FROM_amines_aliphatic_primary_AND_NAS_electrophile.rxn	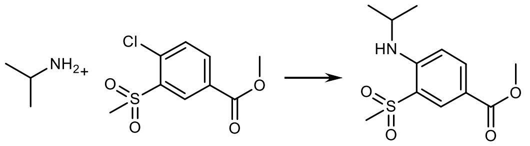
	X.X.X_SNAr_aniline_synthesis_FROM_amines_aliphatic_secondary_AND_NAS_electrophile.rxn	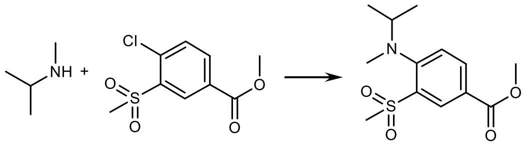
	X.X.X_SNAr_aniline_synthesis_FROM_NAS_electrophile_AND_amines_aliphatic_primary.rxn	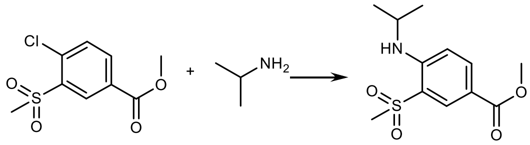
	X.X.X_SNAr_aniline_synthesis_FROM_NAS_electrophile_AND_amines_aliphatic_secondary.rxn	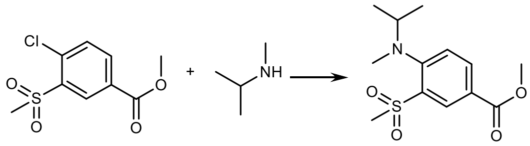
	X.X.X_SnAr_aniline_synthesis_FROM_triazine_chloro_AND_amines.rxn	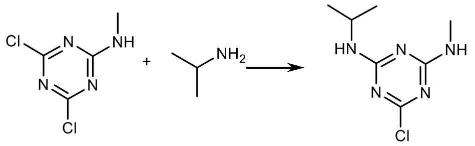
	X.X.X_Carboxylic_acid_+_nitro_condensation_FROM_carboxylic_acids_AND_o_nitro_sec_aniline.rxn	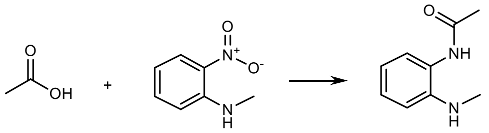
	X.X.X_Carboxylic_acid_+_nitro_condensation_FROM_o_nitro_sec_aniline_AND_carboxylic_acids.rxn	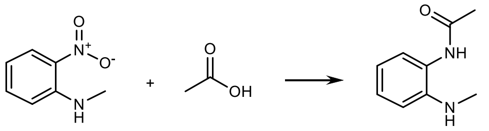

**Supplementary Table 3:** Figures of eDESIGNER deprotection reaction examples		
id	Name	Example
1	3.3.2_Bromo_Sonogashira_coupling_FROM_arylbromide_AND_Null.rxn	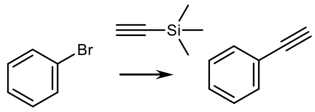
	3.3.4_Iodo_Sonogashira_coupling_FROM_aryliodide_AND_Null.rxn	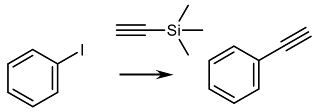
	6.1.1_N-Boc_deprotection_FROM_boc_AND_Null.rxn	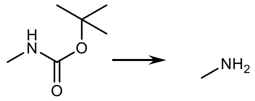
	6.1.6_N-Fmoc_deprotection_FROM_fmoc_AND_Null.rxn	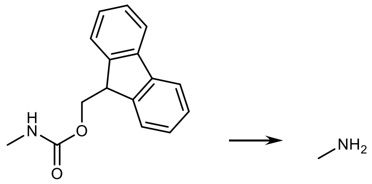
	7.1.1_Nitro_to_amino_FROM_nitro_AND_Null.rxn	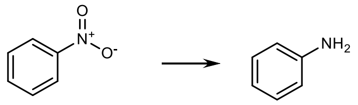
	9.7.61_Ester_hydrolysis_FROM_esters_methyl_ethyl_AND_Null.rxn	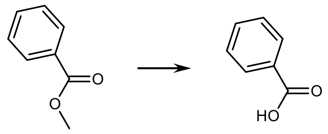
	9.7.8_Amino_to_guanidino_FROM_amines_aliphatic_AND_Null.rxn	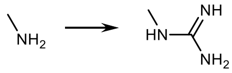
	X.X.X_Triazine_Dichloro_FROM_amines_aliphatic_AND_Null.rxn	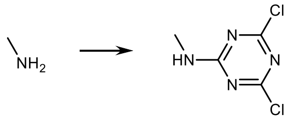
	X.X.X_Esters_methyl_ethyl_WITH_Amines_FROM_aldehydes_AND_Null.rxn	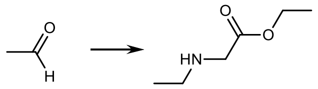
	X.X.X_Nitro_fluoro_FROM_amines_aliphatic_AND_Null.rxn	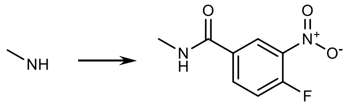
	X.X.X_O_nitro_sec_aniline_WITH_Carboxylic_acid_FROM_amines_aliphatic_primary_AND_Null.rxn	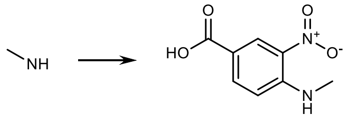
	X.X.X_O_iodo_aniline_FROM_amines_aliphatic_AND_Null.rxn	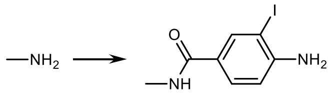

Supplementary method 2: eDESIGNER Code.

2.1.- Functional groups (FGs)

Functional groups are coded as integer indexes. The functional group 0 is defined as *Null Functional Group*, which is added for convenience purposes in the computation.

2.2.- Building Block Types (BBTs)

BBTs are defined as a combination of exactly three functional groups and are coded as a tuple of three numbers, each of these representing the index of one functional group. BBTs are represented also by a sparse vector of 42 dimensions, each representing one possible functional group, except the null FG, and containing the number of occurrences of this functional group in the BBT.

The BBT objects are coded in python as an instance of the BBT class. The attributes of the class are summarized in Supplementary figure 5 of this supplementary information. The main attributes are the *BBT* and *BBT_long* which are python lists describing a condensed and sparse version of the vector listing the FGs combination for this BBT respectively. The *n_compounds* and the associated *n_internal* and *n_external* fields will be used later to ensure that the BBTs incorporated into a library eDESIGN fulfill the pre-defined number of atoms distribution in the final molecules. The integers in these lists represent the total number of building blocks belonging to the BBT with equal or less heavy atom count than a given atom number for each value of that number ranging from 0 to 100. Here it is important to note that, the number of atoms recorded is not the number of atoms calculated directly from the building block smiles, but the number of atoms from the free base or acid smiles corrected by the number contained in the *atom_dif* column in the functional group table (***fg*** spreadsheet in parameters workbook), for each of the FGs belonging to this BBT. This number represents the most probable number of atoms that a building block belonging to this BBT will contribute to the final molecule in the library and we will refer to it as the effective number of heavy atoms.

2.3- Incorporation of building blocks to BBTs

Building blocks that will be later used in the enumeration of real examples are incorporated in files named with the index of the BBT they belong to. There are two files for each BBT, one containing only internally available BBs and the other containing all possible BBs. The number of atoms and rotatable bonds for each BB is calculated by subtracting the *excess_rb* column or adding the *atom_dif* column values to the number of calculated rotatable bonds or number of atoms respectively and eliminating those outside the pre-specified limits. The remaining building blocks are assigned to the corresponding BBTs. When the same free base or acid is repeated in multiple entries from the same or different databases, only one instance of the molecule is stored, with all compound IDs appended to a field in that entry for back tracking. Building blocks are stored in BBT files in increasing order of number of atoms and the number of compounds for each number of atoms is stored in the BBT instance dictionary.

- BBT: List of 3 integers (index of the FG in each position of the BBT, 0 is the index for the null FG)
- BBT_long: List of 43 integers (0 to 3) indicating the presence and number of times ech FG is contained in the BBT
- n_compounds: list on integers indicating the number of unique BBs found in databases for this BBT for each atom number. The number is cumulative so the las entry contains all the compounds found
- n_internal: List of integers indicating the number of unique BBs found in internal databases for this BBT for each atom number. The number is cumulative so the last entry contains all the compounds found
- n_external: List of integers indicating the number of unique BBs found in external databases for this BBT that are not found also in internal databases for each atom number. The number is cumulative so the last entry contains all the compounds found
- min_atoms: The number of atoms of the molecule with least atoms in the BBT
- BBT_name: List of 3 strings indicating the name of the FGs present in the BBT
- BBT_multi : Integer indicating the number of non null FGs in this BBT
- index: Integer indicating the index of this BBT (consecutive number from 0 to number of valid BBTs-1)
- order: Integer indicating a different order appropriate for spotfire visualization
- headpiece: Integer or None. If the BBT can act as headpiece it holds the headpiece index, otherwise is None.
- smiles_example: Smiles string of the one BB assigned to this BBT or None if there are not BBs assigned

Supplementary Figure 5. BBT codification

2.4- Reactions:

Each reaction is coded with a pair of tuples containing two integers each. The first component of the first tuple is the index of the reacting FG being carried by the BBT attached to the DNA (on-DNA). The second number of the first tuple corresponds to the reacting FG being

carried by the incoming BBT, added as a reactant (off-DNA), if such BBT exists; otherwise the second index of the first tuple becomes 0. The second tuple contains the FGs that arise from the reaction, if any. If there are no new FGs formed, the second tuple becomes (0, 0).

There are two main types of reactions: The first type contains reactions that connect two BBTs using an *exposed functional group* from each one. When this happens, in most cases, the original functional groups cancel out and become *no functional groups*. For example, an amidation reaction uses the carboxylic acid functional group (5) and the primary aliphatic amine functional group (21) to attach two building blocks and produces as a result an amide functional group. Since amides are not in the list of eDESIGNER functional groups, the net result is that the reaction produces two new *no functional groups* and the codification of the reaction is (5, 21); (0, 0). Other reactions produce at least one functional group that is included in the eDESIGNER functional groups list. For example, the reductive amination of primary aliphatic amines (21) and aldehydes (1) (Supplementary figure 1, entry b) result in a secondary aliphatic amine functional group (3), which is in the list of functional groups. The codification for this reaction becomes (21, 1); (3, 0).

The second type of reactions comprise the de-protection reactions and the scaffold introduction reactions since none of them involve an off-DNA BBT and, therefore, the second index of the first tuple is always 0. One example of de-protection reaction is the BOC de-protection of aliphatic primary amines (Supplementary figure 4, entry c). The codification of this reaction is (16, 0); (21, 0) since the primary amine BOC-protected is coded with the index 16. The scaffold inclusion reaction is very similar in nature to the de-protection reaction. For example, the introduction of the triazine scaffold by a reaction of a primary aliphatic amine with cyanuric acid is coded as (21, 0); (41, 0). 21 is the index of the primary aliphatic amine functional group and 41 is the index of the dichlorotriazine functional group. Note that, in this case, and in contrast to the BOC de-protection reaction, additional mass is added to the on-DNA BBT. However, the mass does not come from a BBT but from a reactant (in other words, the incorporated chemical matter is not variable, it is the same for all the molecules in the library) and therefore, for technical implementation reasons, the reaction is grouped with the de-protection reactions rather than to the connection reactions. This has implications that are explained later.

2.5.- Creation of eDESIGNS:

Supplementary figure 6 in this supplementary information, lists the encoding of an eDESIGN. The eDESIGN, similarly to the BBT object, is coded as a class and each individual eDESIGN is an instance of this class. The collection of eDESIGNS is then stored in a list of eDESIGN instances. The eDESIGN dictionary stores the BBTs used, the deprotections used to activate functional groups and to incorporate scaffolds, the reactions used to connect BBTs and the connectivity (topology) of the design. Thus, the field *n_cycles* holds the information of how many BBTs the design incorporates (excluding the headpiece). The field *bbts* contain the index of the BBT that is incorporated in each cycle (including the headpiece as cycle 0). The field *fgs* contains the list of non-null FG indexes that each design holds at any given step.

At each cycle, eDESIGNER will attempt a de-protection or scaffold incorporation and then conduct a reaction where a new BBT is incorporated. The indexes for these reactions and de-protections are stored in the *reactions* and *deprotections* fields respectively. For each FG in the *fgs* field, a code representing its origin (when and how it was incorporated to the eDESIGN) is stored in the field *fg_sources*. The source of an FG can be either a BBT, a reaction or a deprotection. For example, a reductive amination of a primary amine creates the secondary amine FG, an ester hydrolysis creates the acid FG and any FG can be incorporated as a part of a BBT. The topology fields track the source of the functional group that was used to attach each of the BBTs or to conduct a deprotection in the eDESIGN. There are two types of topology fields. The btopology tracks the source of the FG used to attach each BBT and the dtopology tracks the source of the FGs that are used to conduct each deprotection.

The initial list of eDESIGNS is created with the six headpieces that are incorporated by adding all non-null FGs to the *fgs* field and updating sources and topology. Then, the first de-protection reaction is attempted by matching the first index of the first tuple of the reaction (the on-DNA index) with every index in the *fgs* field of the eDESIGN. Whenever a match is found a new eDESIGN is created by cloning the current design. Then, the new design is checked out for reaction incompatibilities (vide infra) and, if it survives, it is appended to the list of designs after adjusting all the eDESIGN fields including the *fgs* field with the information from the reaction. The original eDESIGN is always kept in the list of designs when incorporating deprotections. The net result of the incorporation of a de-protection step is a new set of eDESIGNS from each eDESIGN, where the original one is kept and all possible compatible de-protections (or scaffold incorporations) are executed, one at a time, each one generating a new design.

Each reaction in the *reaction* and *deprotection* parameters tables (parameters workbook) has an index (column *n*), which is incorporated into the eDESIGN *fgs* field. The pair of tuples coding the reaction are listed in the columns *fg_input_on_off* and gf_output_on_off. The first tuple describes the input FGs. Since there is not an incoming BBT in a deprotection reaction, the second element of the first tuple is always 0 for deprotections. The second tuple describe the FGs that are added to the design, if any. The column *excluded_on* lists all the functional groups, that are originally in the incoming eDESIGN, which are not compatible with this specific reaction. The *excluded_off* field contains the list of FGs that are part of the incoming BBT that are incompatible with the reaction. Again, this is set to -1 for deprotections since there is not incoming BBT for these reactions. The production column is added as an extra functionality so the user can define parametrically a subset of reactions to use in the study. In our case, we divided the reactions in two categories, the first containing reactions previously used in a library production and the second containing reactions only validated experimentally but not used in production at the date of writing this manuscript.

Once the first deprotection reaction is added, the next task of eDESIGNER is to add the first BBT with a connection reaction. The process is similar to the one described above for the de-protection reaction but more combinatorial in nature. Each eDESIGN from the current list is evaluated but, contrary to what happened with the de-protection reaction, the original eDESIGN is not appended to the new list because only the designs able to grow can be incorporated to the list. A selection of all available BBTs (available means that there is at least one building block assigned to the BBT, 262 in this implementation) are attempted to react with each eDESIGN. For each BBT – eDESIGN combination all the pairs comprised of one FG from the eDESIGN and one FG from the BBT are enumerated, and for each pair a search is performed in the *fg_input_on_off* field of the connection reactions list. If a match is found, after checking for incompatibilities among FGs

in the eDESIGN and incoming BBT, the original eDESIGN is cloned, the incoming BBT appended, the FGs of the incoming BBT added to the design and the reacting FGs modified appropriately according to the reaction code. Then, a further check is performed: for an eDESIGN to be able to grow, it must contain at least one non-null functional group in the *fgs* field. All eDESIGNs that don't fulfill these criteria are eliminated unless the current cycle is the last one. For eDESIGNs that survive the checks after each cycle, eDESIGNER updates all their fields.

- id: Integer unique for each eDESIGN
- n_cycles: Integer representing how many cycles contain the design
- bbts: List of integers representing the indexes of the BBTs included in each cycle
- fgs: List of integers representing the indexes of the functional groups exposed for reaction in a design
- reactions: List of integers representing the indexes of reactions used to incorporate each BBT
- deprotections: List of integers representing the indexes of de-protection reaction used before each cycle
- fg_sources: list of integers representing the source of each funtional group*
- btopology: list of integers representing the topology of the BBT added at each cycle (the *fg_sources* value for the FG that is used to connect this BBT)*
- dtopology: list of integers representing the topology of the deprotection reaction used in each cycle (the *fg_sources* value for the FG that each deprotection reaction acts on in each cycle. If a cycle does not incorporate a deprotection the *dtopology* value for that cycle becomes -1)*
- lib_id: tuple formend by concatenation of the index for the headpiece(*bbts[0]*), the number of cycles (*n_cycles*), the indexes of the enumeration reactions used, the index of the enumeration deprotections, the *btopology* elements and the *dtopology* elements. This tuple is unique for each lib_DESIGN and will become the id of the lib_DESIGN formed by grouping compatible eDESIGNS.
- min_atoms: Integer representing the number of atoms of the molecule with the least toms in the design at the current cycle.

* -1 = DNA, 0 = Headpiece, 1 = deprotection at cycle 1, 2 = reaction at cycle 1, 3 = BBT at cycle 1, 4 = deprotection at cycle 2 , 5 = reaction at cycle 2, 6 = BBT at cycle 2, 7 = deprotection ac cycle 3, 8 = reaction at cycle 3, 9 = BBT at cycle 3

Supplementary Figure 6: eDESIGN codfication

2.6- Creation of libDESIGNS:

The libDESIGN is coded as a python class and instantiated for each library. The attributes in the instance dictionary are described in Supplementary figure 7 in this supplementary information.

Before combining designs, all eDESIGNs are tagged using a tuple of integers coming from specific data in the eDESIGN and named *lib_id*. This tuple comprises the index of the BBT serving as a headpiece, the *enum_indexes* for all the construction reactions, the *enum_indexes* for all the de-protection reactions, and the *topology indexes* stored in the eDESIGN. All eDESIGNS that contain the same *lib_id* can be combined into a single design and produced experimentally as a single library. Therefore, once the *lib_id* is set for all eDESIGNS the libDESIGNs are constructed by combining all the eDESINGS with the same *lib_id*.

The libDESIGN *deprotections* and *reactions* fields are based on the eDESIGN *reactions* and *deprotections* fields and specifically the values of the column *enum_index* parameters in the eDESIGN. These must be common for all the eDESIGNS corresponding to a libDESIGNs by definition. The *scaffold_reactions* field is extracted from the *deprotections* field. A deprotection reaction is determined to produce a scaffold when the number of heavy atoms produced by the reaction is positive. This number is taken from the *atom_diff* field for each specific reaction and the new value is stored as the *enum_index* for that specific reaction, so it is common for all eDESIGNs being combined. The *bbts* field of the libDESIGN contain a list for each cycle in the original eDESIGNs being each member of the list the index of the BBT for that eDESIGN at that cycle. Once the lists are created, the duplicated BBT indexes at each cycle are removed, so each BBT index appears only once for each cycle.

The final step is to filter all libDESIGNs that do not reach a minimum number of compounds per design. We used the value *n_int* and a predefined parameter to filter out these libDESIGNs. The predefined parameter is the number of atoms that corresponds to the median of the desired distribution, so the result obtained by the method described above gives the maximum number of compounds for this libDESIGN containing a median that is equal or less than the predefined parameter. Once the number corresponding to the median is known the total number of compounds is straightforward to calculate; what is not known at this time is the number of atoms of the largest molecule that would give rise to this number of molecules at percentile 100. In order to calculate this number, and the maximum number of atoms coming from each cycle, the procedure described above is repeated for an increasing number of atoms until the calculated number of molecules in the design reaches the percentile 100, or the total number of atoms reaches a pre-specified parameter representing the maximum number of atoms allowed for a molecule in the lib_DESIGN. At this point the maximum number of atoms coming from each cycle is stored in the field *best_index* of the lib_DESIGN and the *int_limits*, *all_limits*, *n_int* and *n_all* calculated and stored using this value.

- Id: Integer index that is unique for each macroDESIGN.
- lib_id: tuple of integers (headpiece, reactions, deprotections and topology) that uniquely identifies a macroDESIGN.
- design_id:
- n_cycles: Integer representing how many cycles contain the macroDESIGN.
- headpeice: Building block type of the headpiece
- bbts: List of lists of integers representing the indexes of the BBTs included in each cycle for each design in the macroDESIGN
- int_limits: list of integers representing the number of compounds that have to be taken from each individual BBT smiles file, to add up for a cycle (from internal BBTs files)
- all_limits: List of integers representing the number of compounds that have to be taken from each individual BBT smiles file to add up for a cycle (from all BBTs files)
- n_int: Number of compounds in the library taking only internal compounds
- n_all: Number of compounds in the library taking both internal and external compounds
- reactions: List of integers representing the indexes of **enumeration-reactions** used to incorporate each BBT
- deprotections: List of integers representing the indexes of internal **enumeration-deprotection** reactions used before each cycle
- scaffold_reactions: List of integers representing the indexes of the **original-reactions** that incorporate an scaffold (used to calculate the number of atoms coming from added scaffold)
- best_index: list of integers representing the best combination of maximum atoms incorporated in each cycle to meet the heavy atom distribution requirements
- eliminate: (TRUE or FALSE): Whether to eliminate or not this macroDESIGN from the list

Supplementary Figure 7: libDESIGN codification

Once the libDESIGN is created, the next step is to calculate the *int_limits*, *all_limits* and *best_index* fields. The value of those fields determines the heavy atom distribution of final molecules in the libDESIGN and are calculated in such a way that the number of molecules in the libDESIGN is maximized while the desired heavy atom distribution is maintained.

As described before, the *bbts* field contains the list of BBT indexes corresponding to the BBTs that can be mixed in that specific cycle. The *int_limits* and *all_limits* fields have the same structure (a list of integers for each cycle), but their values represent how many building blocks must be taken from each BBT smiles file (starting from the beginning) to construct the final file containing building blocks for that cycle. Since the compounds in the smiles file corresponding to each BBT were sorted in ascending order by heavy atoms count, the number of compounds taken from each individual file will determine the heavy atom distribution of the library. The reason that there are two different fields is because we have created two versions of each BBT smiles file, one containing only internally available building blocks (and thus accessible immediately) and the other containing both internally and externally compounds.

The *best_index* field is the key field to determine the heavy atom distribution since the *int_limits* and *all_limits* fields are both derived from it. The *best_index* field represents the maximum number of heavy atoms that is allowed for a BBT in each cycle. This is a list of integers (one per cycle), and it is the value that maximizes the number of compounds in a libDESIGN while keeping the desired heavy atom distribution.

The number of heavy atoms in a molecule belonging to a libDESIGN depend on three values only: the number of atoms supplied by the headpiece, the number of atoms contained in the added scaffolds and the number of atoms supplied by the building blocks. The first value is taken as a parameter. It is worth noting that the headpiece (and attached DNA) is a huge molecule, that we largely ignore for atom counting purposes and solely focus on what it is going to be resynthesized off-DNA after finding actives. The decision of what is the exact portion of the headpiece added to the molecule to be re-synthesized is left to the medicinal chemist. In practice it is usually a small piece, therefore for the purposes of this study we have used 4 atoms as the parameter. The second value (the number of heavy atoms supplemented as scaffolds) is taken from the *deprotection* parameters table (parameters workbook) for each specific libDESIGN. After compiling all the de-protection reactions that increase molecular mass (stored in the libDESIGN field *scaffold_reactions*) the number of added atoms is computed by adding the *atom_diff* parameter. Since the first two values are constant for each libDESIGN, the optimization focuses on the third set of values (atoms that come from each building block). The method used is to enumerate all possible combinations of number of atoms coming from each cycle where the sum of those atoms plus the atoms coming from the headpiece and scaffolds add up to one pre-specified number (vide infra). Then, for each combination, the total number of molecules with equal or lower number of heavy atoms is computed. This is done using the field *n_internal* in the BBT dictionary corresponding to each BBT in the lib_DESIGN. The combination that gave the highest number of compounds was the one selected and stored along the number of compounds that it would produce.

2.7.- Library compound enumeration:

Library enumeration uses instructions in libDESIGN configuration files. Typically, each enumeration instruction set starts with the introduction of a headpiece chemical structure represented in smiles format. Instructions for each synthetic step or cycle follow including

reactions introducing appropriate building block sets and any necessary deprotection reactions. In the case of the former type of reactions necessary building block sets are provided through the libDESIGN file. Standard functional group deprotections are also invoked at the conclusion of the enumeration process to ensure that molecules containing matching protecting groups are properly processed. Note that molecules not containing protecting groups are left intact.

A sample of a libDESIGN configuration file for eDesigner 2-cycle library number 411 is shown in Supplementary figure 8 below. The enumeration is first instructed on how to build the building block files C1.smi and C2.smi that contain building blocks for each cycle. This is performed by picking a molecules from a number of files representing BBTs used in each cycle (format <bbt_file.smi>:number_of_molecules_to_include). The library initiates with an on-DNA substructure indicated at line starting with code word 'START'. Each subsequent step starts with the code word 'AND' and ends with symbol '|'. Within each step the operation to be performed is always described by a reaction file name followed with the necessary reactants if required. Step 1 introduces a scaffold by the reaction of the amine in the headpiece with cyanuric acid. This reaction generates a dichlorotriazine functional group that is used to perform a nucleophilic aromatic substitution with phenols contained in the file C1.smi. The third step is a second nucleophilic aromatic substitution, in this case with a collection of amines contained in the file C2.smi. The last steps are a set of boc and fmoc deprotections and ester hydrolysis to ensure all possible remaining functional groups in the molecules are deprotected.

Supplementary figure 9 presents an example libDESIGN configuration file for 3-cycle library 1273. Supplementary figure 10 presents an example reaction file for amide formation (2.1.2_Carboxylic_acid_+_amine_condensation_FROM_amines_AND_carboxylic_acids).

```
# Start enumeration instructions
# Design number 411
# Design fingerprint (2, 3, 11, 68, 8, 0, -1, 1, 5, -1, 0, -1)
# Design scope 411.ALL
# Design size 2584050
# Design number of cycles 2
# MAKE C1.smi WITH
{'9.smi':686,'90.smi':6,'188.smi':23,'272.smi':39,'309.smi':94,'310.smi':12,'311.smi':8,'313.smi':96,'314.smi':330,'320.smi':41,'322
.smi':4,'2808.smi':9,'2809.smi':16,'3740.smi':1,'3744.smi':10,'3745.smi':35,'4213.smi':15,'4214.smi':46,'4250.smi':2,'4288.s mi':1,'
4302.smi':24}
# MAKE C2.smi WITH
{'3.smi':413,'21.smi':686,'22.smi':1,'84.smi':22,'102.smi':32,'124.smi':12,'127.smi':1,'130.smi':49,'132.smi':74,'133.smi':44,'137.s
mi':2,'200.smi':29,'434.smi':113,'473.smi':102,'498.smi':93,'545.smi':2,'565.smi':17,'566.smi':3,'586.smi':5,'588.smi':4,'590.smi':1
,'1361.smi':1,'1658.smi':3,'2968.smi':6,'5311.smi':9,'5336.smi':1}START: [13CH3]OCCNC core
AND:
<reactions_folder>/X.X.X_Triazine_Dichloro_FROM_amines_aliphatic_AND_Null.rxn
|
AND:
<reactions_folder>/1.7.11_SNAr_ether_synthesis_FROM_triazine_chloro_AND_phenols.rxn||file=<compounds_folder>/C1.smi
|
AND:
<reactions_folder>/X.X.X_SnAr_aniline_synthesis_FROM_triazine_chloro_AND_amines.rxn||file=<compounds_folder>/C2.smi
|
AND:
<reactions_folder>/6.1.1_N-Boc_deprotection_FROM_boc_AND_Null.rxn
NOOP
|
AND:
<reactions_folder>/6.1.1_N-Boc_deprotection_FROM_boc_AND_Null.rxn
NOOP
|
AND:
<reactions_folder>/6.1.6_N-Fmoc_deprotection_FROM_fmoc_AND_Null.rxn
NOOP
|
AND:
<reactions_folder>/6.1.6_N-Fmoc_deprotection_FROM_fmoc_AND_Null.rxn
NOOP
|
AND:
<reactions_folder>/9.7.61_Ester_hydrolysis_FROM_esters_methyl_ethyl_AND_Null.rxn
NOOP
|
AND:
<reactions_folder>/9.7.61_Ester_hydrolysis_FROM_esters_methyl_ethyl_AND_Null.rxn
NOOP
# End enumeration instructions
```

Supplementary Figure 8. lib_DESIGN number 441 in configuration file used for the enumeration of a DEL library. Comment lines begin with symbol ‘#’

```
# Start enumeration instructions
# Design number 1273
# Design fingerprint (3, 21, 7, 49, 27, 0, 0, 6, -1, 0, 5, 7, -1, -1, -1, 9)
# Design scope 1273.ALL
# Design size 41374476
# Design number of cycles 3
# MAKE C1.smi WITH {'15.smi':28,'459.smi':8,'460.smi':30}
# MAKE C2.smi WITH {'55.smi':121,'1064.smi':4,'1065.smi':3,'1068.smi':13}
# MAKE C3.smi WITH
{'3.smi':1172,'21.smi':1672,'22.smi':10,'84.smi':46,'102.smi':56,'127.smi':2,'130.smi':156,'132.smi':227,'133.smi':169,'136.smi':8,'
137.smi':12,'139.smi':2,'316.smi':2,'434.smi':250,'473.smi':189,'498.smi':287,'544.smi':7,'545.smi':19,'565.smi':63,'566.smi' :33,'5
86.smi':7,'588.smi':5,'590.smi':1,'1361.smi':1,'1658.smi':6,'2063.smi':7,'2064.smi':6,'4284.smi':1,'4298.smi':6,'5311.smi':12,'5336
.smi':11,'5426.smi':1}
START: [13CH3]OCCN core
AND:
<reactions_folder>/1.3.8_Fluoro_N-arylation_FROM_amines_AND_nitro_fluoro.rxn||file=<compounds_folder>/C1.smi
|
AND:
<reactions_folder>/4.1.45_Benzimidazole_synthesis_FROM_o_nitro_sec_aniline_AND_aldehydes.rxn||file=<compounds_folder
>/C2.smi
|
AND:
<reactions_folder>/9.7.61_Ester_hydrolysis_FROM_esters_methyl_ethyl_AND_Null.rxn
|
AND:
<reactions_folder>/2.1.2_Carboxylic_acid_+_amine_condensation_FROM_carboxylic_acids_AND_amines.rxn||file=<compound
s_folder>/C3.smi
|
AND:
<reactions_folder>/6.1.1_N-Boc_deprotection_FROM_boc_AND_Null.rxn
NOOP
|
AND:
<reactions_folder>/6.1.1_N-Boc_deprotection_FROM_boc_AND_Null.rxn
NOOP
|
AND:
<reactions_folder>/6.1.6_N-Fmoc_deprotection_FROM_fmoc_AND_Null.rxn
NOOP
|
AND:
<reactions_folder>/6.1.6_N-Fmoc_deprotection_FROM_fmoc_AND_Null.rxn
NOOP
|
AND:
<reactions_folder>/9.7.61_Ester_hydrolysis_FROM_esters_methyl_ethyl_AND_Null.rxn
NOOP
|
AND:
<reactions_folder>/9.7.61_Ester_hydrolysis_FROM_esters_methyl_ethyl_AND_Null.rxn
NOOP
# End enumeration instructions
```

Supplementary Figure 9. lib_DESIGN number 1273 in configuration file used for the enumeration of a DEL library. Comment lines begin with symbol '#'

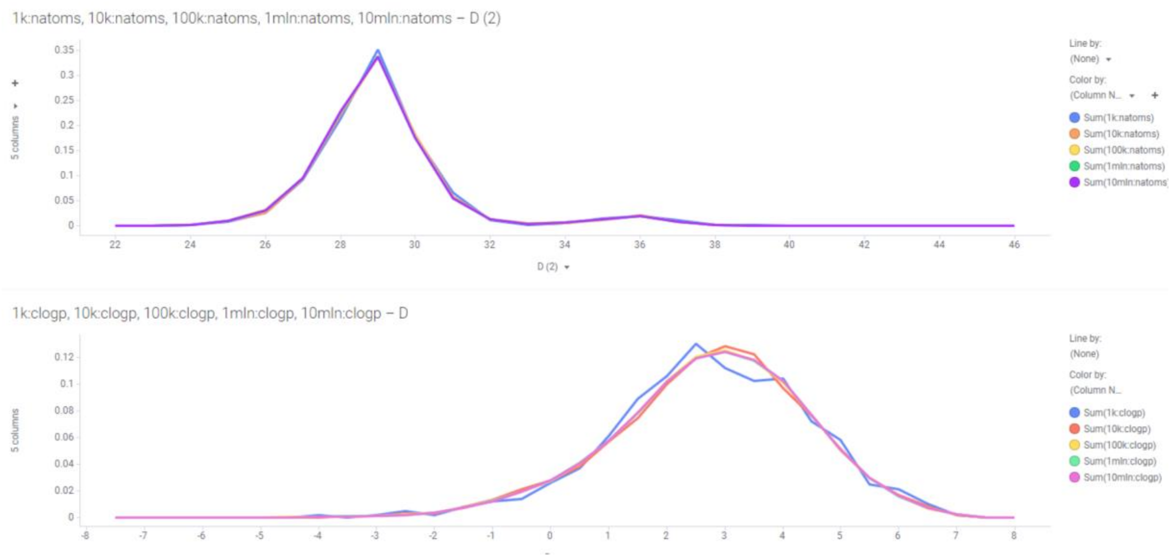

Supplementary Figure 10. Distributions of number of atoms (top) and clogP (bottom) for 5 random subsets of 2-cycle libDESIGN 411. The 5 subsets consist of 1k, 10k, 100k, 1mln and 10mln compounds. Note that the distributions of all sets are practically indistinguishable with the exception of the 1k subset.

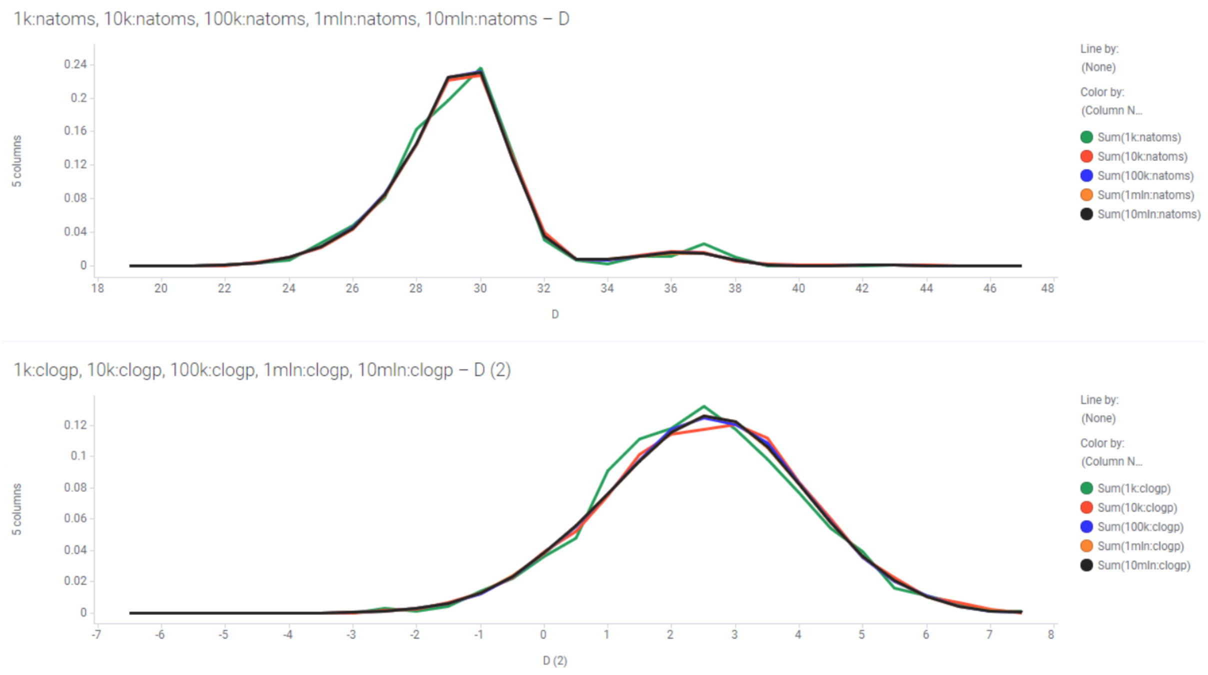

Supplementary Figure 11. Distributions of number of atoms (top) and clogP (bottom) for 5 random subsets of 3-cycle libDESIGN 1273. The 5 subsets consist of 1k, 10k, 100k, 1mln and 10mln compounds. Note that the distributions of all sets are practically indistinguishable with the exception of the 1k subset.

```
(0 Reaction
 (A C Comment "2.1.2_Carboxylic_acid_+_amine_condensation_FROM_amines_AND_carboxylic_acids")
 (0 Scaffold
   (A C smarts "[ND1H2]-[CX4]||[ND2H]([CX4])[CX4]||[ND1H2]-a||[ND2H]([CX4])-a||[ND2H](a)-a")
 )
 (1 Sidechain
   (A C smarts "[C](=O)([OH])")
   (A I remove_atom 2)
   (A I join (0 0))
 )
)
```

Supplementary Figure 12: Sample reaction description file in LillyMol format for reaction type: 2.1.2_Carboxylic_acid_+_amine_condensation_FROM_amines_AND_carboxylic_acids.rxn. The reaction instructions connects “scaffold” building blocks with id 0 to “sidechain” building blocks with id 1. The scaffold building blocks must contain an aliphatic or aromatic primary or secondary amine as represented by the smarts within the bracketed description of “Scaffold” description. The sidechain building blocks must contain a carboxylic acid as represented by the smarts within the bracketed description of “Sidechain” description. Leaving atoms are indicated by the “remove_atom” directive. Bond joins are indicated by the “join” directive.

A full listing of the reactions used by eDesigner can be found at https://github.com/EliLillyCo/LillyMol section contrib, eDesigner paper.

Supplementary method 3: Sample Commands.

The following commands can be found in the open source LillyMol github project available at: https://github.com/EliLillyCo/LillyMol

3.1- Building Block Annotation file preparation example:

```
tsubstructure -v -q F:fgqueries -A D -a train.smi > train_profile.txt
    (generate a functional group annotation table (-a) for structures in train.smi using standard functional group annotation queries listed in file fgqueries(-q); use daylight aromaticity (-A); verbose mode (-v))
```

The list of functional group queries and the 'fgqueries' file can be found in the contrib folder of the LillyMol github project.

3.2- libDESIGN enumeration example:

```
molecular_transformations -N 10000 -S sample_design -m RMX -z i -M RMX -Z -W rxsep='>>' -W rgsep='+' -A D -u -T sample_design.config
    (read sample_design.config configuration file (-T) and enumerate 10000 structures (-N); store the enumerated structures in file with stem sample_design; use Daylight aromaticity rules (-A D), one embedding per start atom (-u); ignore non reacting molecules (-z i); ignore non reacting sidechains (-Z); ignore molecules with multiple substructure matches (-m RMX); ignore sidechains with multiple substructure matches (-M RMX); place '>>' between reaction steps (-W rxsep='>>'); place '+' between reagents (-W rgsep='+')
```

3.3- Fingerprint generation command example:

```
iwfp train.smi > train.gfp
    (calculate hashed path-based fingerprints for structures in train.smi
```

3.4- Near neighbor calculation command example:

```
gfp_nearneighbours -p pubchem.gfp -n 2 -T 0.5 train.gfp > train.nn
        (compare fingerprints in train.gfp (needles) against fingerprints in fingerprint set pubchem.gfp (haystack, -p); retrieve 2 nearest neighbours for each needle in train.gfp (-n 2); discard distances greater than 0.5 (-T 0.)
```

3.5- Spread calculation command example:

```
gfp_spread_v2 -A haystack.gfp train.gfp > train.spd
        (compute the spread distance of fingerprints in train.gfp. Bias away from fingerprints in the reference set haystack.gfp (-A))
```

Supplementary Notes

Supplementary note 1: Reaction utilization.

Supplementary figures 13 and 14 summarize the number of instances each reaction has been used in the selected 2-cycle and 3-cycle libDESIGNS respectively. Supplementary figures 15 and 16 summarize the number of instances each deprotection / scaffold incorporation has been used in the selected 2-cycle and 3-cycle libDESIGNS respectively.

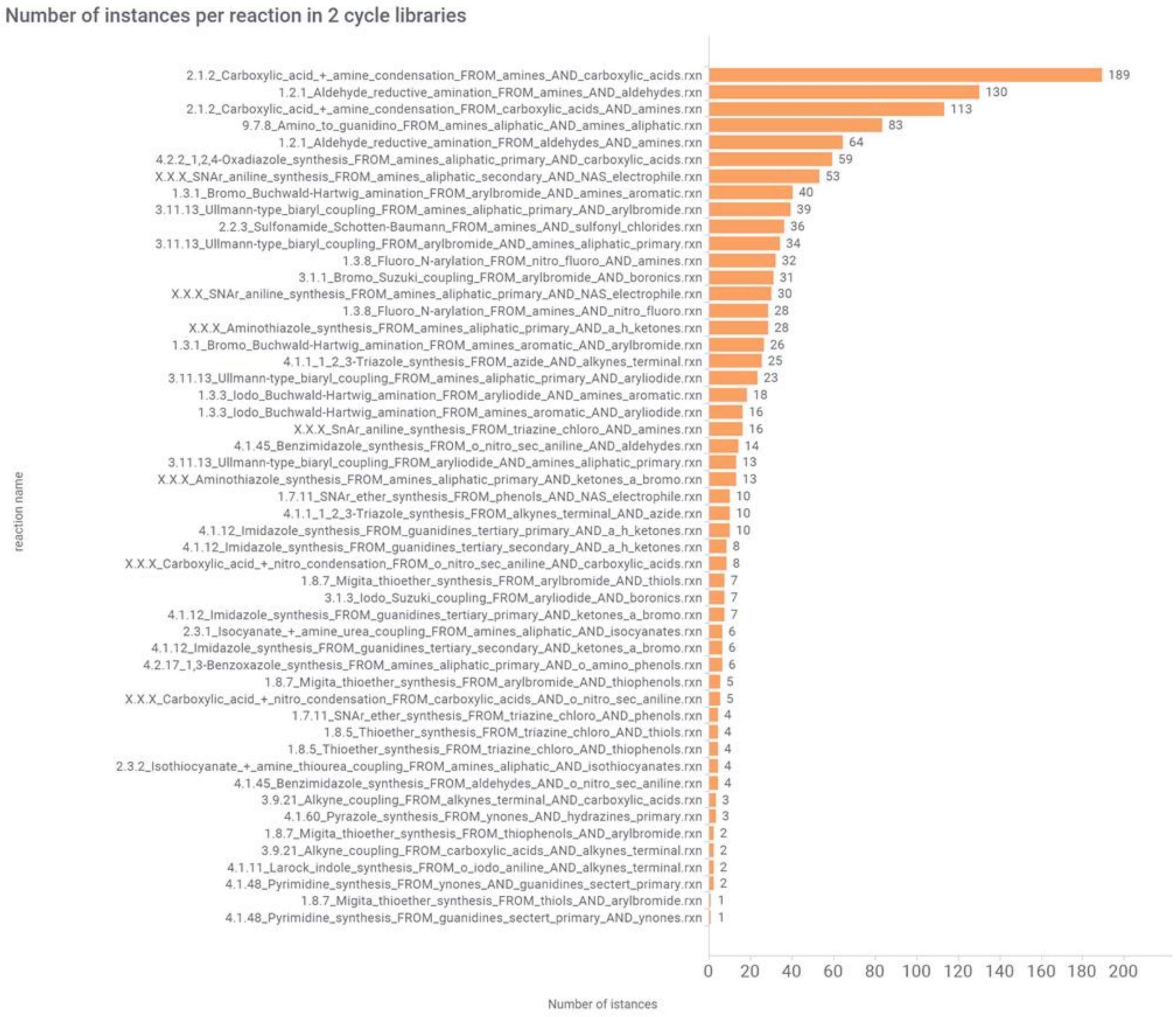

Supplementary Figure 13. BBT incorporation reaction analysis for 2 cycle libraries

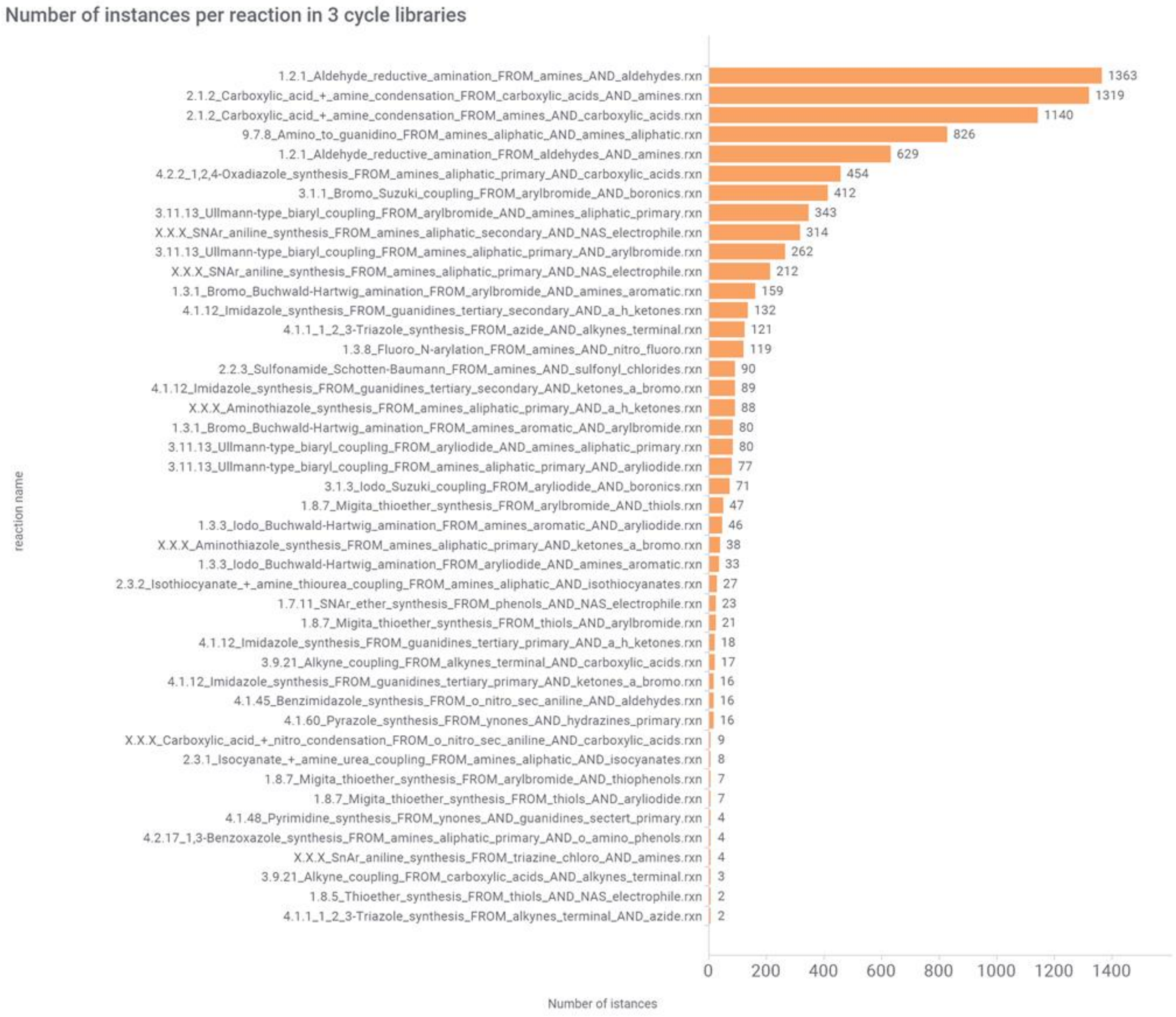

Supplementary Figure 14. BBT incorporation reaction analysis for 3 cycle libraries

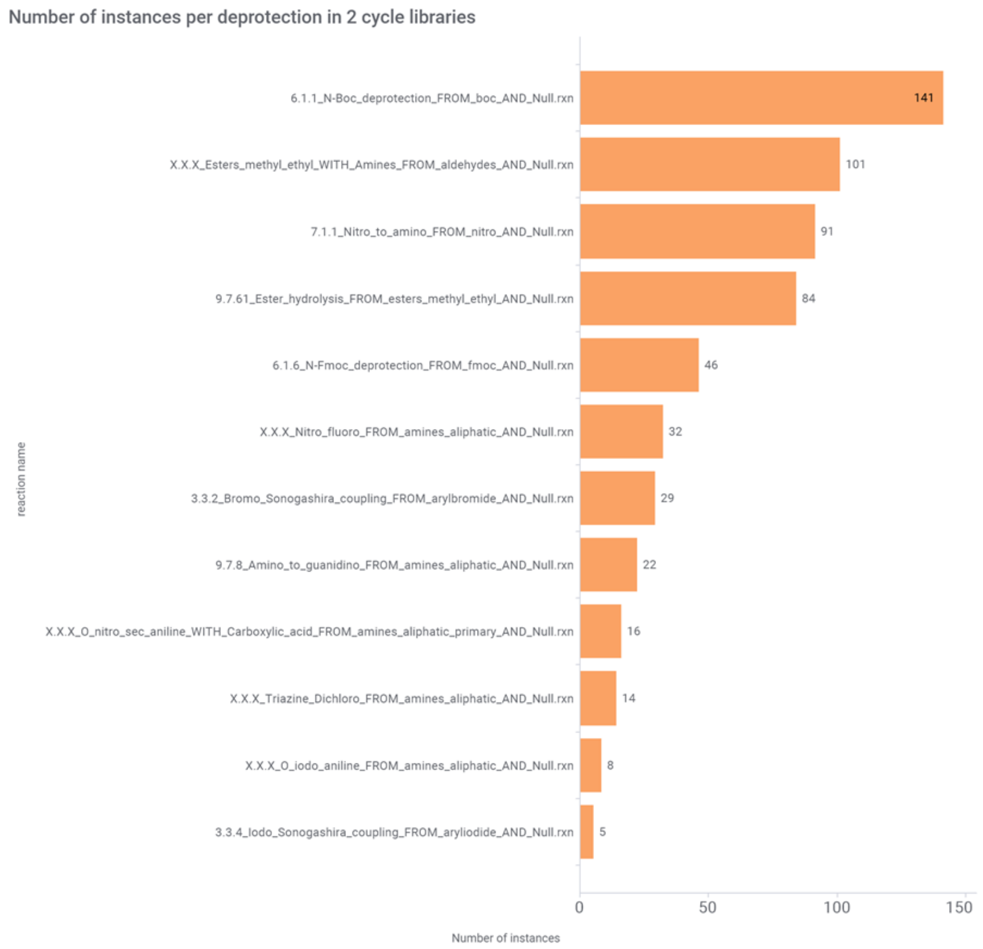

Supplementary Figure 15. deprotection reaction analysis for 2 cycle libraries

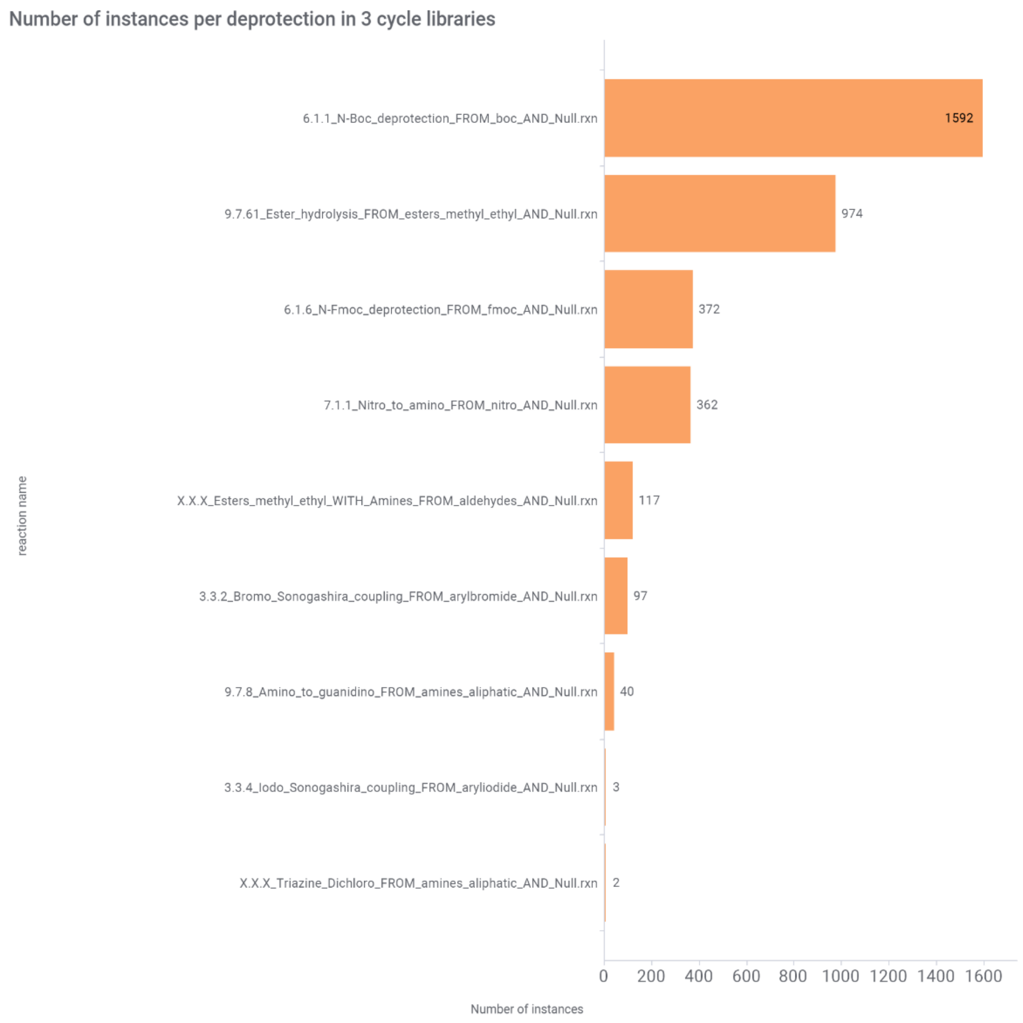

Suppmentary Figure 16. deprotection reaction analysis for 3 cycle libraries

Supplementary note 2: Spread design analysis.

Supplementary figure 17 presents spread design analysis on the 3-cycle library (X) sets enumerated with internal BBs. Library spread design rank-orders libraries by diversity to a reference set in this case the ADEL and LDC compound sets. As shown, 3-cycle libDESIGNs have spread values ranging from 0.31 (most diverse from reference sets) to 0.17.

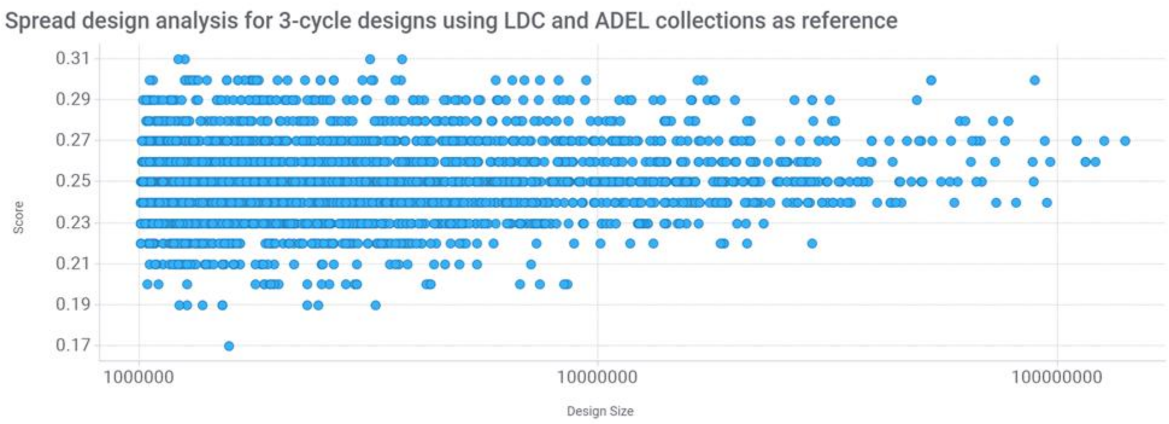

Supplementary Figure 17. Spread design analysis for 3-cycle libDESIGNs
